# Efficacy, Safety and Predictive Biomarkers of Oncolytic Virus Therapy in Solid Tumors: A Systematic Review and Meta-Analysis

**DOI:** 10.3390/vaccines13101070

**Published:** 2025-10-20

**Authors:** Mohamed El-Tanani, Syed Arman Rabbani, Mohamed Anas Patni, Rasha Babiker, Shakta Mani Satyam, Imran Rashid Rangraze, Adil Farooq Wali, Yahia El-Tanani, Thantrira Porntaveetus

**Affiliations:** 1 RAK College of Pharmacy, RAK Medical and Health Sciences University, Ras Al Khaimah 11172, United Arab Emirates; arman@rakmhsu.ac.ae (S.A.R.); farooq@rakmhsu.ac.ae (A.F.W.); 2 RAK College of Medical Sciences, RAK Medical and Health Sciences University, Ras Al Khaimah 11172, United Arab Emirates; mohamedanas@rakmhsu.ac.ae (M.A.P.); rashababiker@rakmhsu.ac.ae (R.B.); satyam@rakmhsu.ac.ae (S.M.S.); imranrashid@rakmhsu.ac.ae (I.R.R.); 3 Royal Cornwall Hospital Trust, NHS, Truro TR1 3LJ, UK; y.el-tanani@nhs.net; 4 Center of Excellence in Precision Medicine and Digital Health, Department of Physiology, Faculty of Dentistry, Chulalongkorn University, Bangkok 10330, Thailand; thantrira.p@chula.ac.th

**Keywords:** oncolytic virus therapy, solid tumors, efficacy, safety, predictive biomarkers, tumor mutational burden, immune microenvironment, gut microbiome, CD8^+^ T-cell infiltration

## Abstract

**Background:** Oncolytic virus (OV) therapy couples direct tumor lysis with systemic immune priming, yet clinical benefit remains heterogeneous and the predictive biomarker landscape is poorly defined. We undertook a systematic review and meta-analysis to quantify the efficacy and safety of OV therapy in solid tumors and to synthesize current evidence on response-modulating biomarkers. **Methods:** Following PRISMA 2020 guidelines, MEDLINE, Embase, Cochrane CENTRAL, ProQuest and Scopus were searched from inception to May 2025. Phase II–III randomized trials of genetically engineered or naturally occurring OV reporting objective response rate (ORR), progression-free survival (PFS), overall survival (OS) or biomarker data were eligible. Hazard ratios (HRs) or odds ratios (OR) were pooled with random-effects models; heterogeneity was assessed with I^2^ statistics. Qualitative synthesis integrated genomic, immunologic and microbiome biomarkers. **Results:** Thirty-six trials encompassing around 4190 patients across different tumor types met inclusion criteria. Compared with standard therapy, OV-based regimens significantly improved ORR nearly three-fold (pooled OR = 2.77, 95% CI 1.85–4.16), prolonged PFS by 11% (HR = 0.89, 95% CI 0.80–0.99) and reduced mortality by 16% (OS HR = 0.84, 95% CI 0.72–0.97; I^2^ = 59%). Benefits were most pronounced in melanoma (ORR 26–49%; OS HR 0.57–0.79) and in high-dose vaccinia virus for hepatocellular carcinoma (HR = 0.39). Grade ≥ 3 adverse events were not increased versus control (risk ratio 1.05, 95% CI 0.89–1.24); common toxicities were transient flu-like symptoms and injection-site reactions. Biomarker synthesis revealed that high tumor mutational burden, interferon-pathway loss-of-function mutations, baseline CD8^+^ T-cell infiltration, post-OV upregulation of IFN-γ/PD-L1, and favorable gut microbial signatures correlated with response, whereas intact antiviral signaling, immune-excluded microenvironments and myeloid dominance predicted resistance. **Conclusions:** OV therapy confers clinically meaningful improvements in tumor response, PFS and OS with a favorable safety profile. Integrating composite genomic–immune–microbiome biomarkers into trial design is critical to refine patient selection and realize precision viro-immunotherapy. Future research should prioritize biomarker-enriched, rational combination strategies to overcome resistance and extend benefit beyond melanoma.

## 1. Introduction

Cancer remains a leading cause of global mortality despite significant advancements in diagnostic modalities and therapeutic strategies [1]. Conventional approaches such as surgery, chemotherapy and radiotherapy, while foundational to oncology care, often suffer from limitations including systemic toxicity, lack of tumor selectivity and resistance development [2,3,4]. These limitations have prompted a paradigm shift toward immunotherapy, which leverages the host immune system to achieve durable tumor control [5]. Among emerging immunotherapeutic strategies, oncolytic virus (OV) therapy has gained prominence due to its unique dual mechanism: direct tumor cell lysis and subsequent activation of systemic antitumor immunity [6,7].

Oncolytic viruses—either naturally occurring or genetically engineered—selectively replicate within cancer cells, causing immunogenic cell death while sparing healthy tissues [8,9,10]. This lytic activity not only destroys tumor cells but also releases tumor-associated antigens (TAAs), damage-associated molecular patterns (DAMPs), and pathogen-associated molecular patterns (PAMPs), which collectively trigger innate and adaptive immune responses [11]. Genetic engineering of OV to express immunostimulatory transgenes, such as GM-CSF or IL-12, further enhances their ability to convert immunologically “cold” tumors into “hot” tumors with robust T cell infiltration [12,13,14,15].

Clinical development of OV therapy has accelerated in recent years. Talimogene laherparepvec (T-VEC), a modified herpes simplex virus, has been approved by the U.S. FDA for advanced melanoma, paving the way for other candidates including adenovirus (ONYX-015), reovirus (pelareorep), vaccinia virus (Pexa-Vec), and seneca valley virus (NTX-010) [16,17,18,19,20,21]. While many of these agents have demonstrated encouraging antitumor responses in early-phase trials, clinical benefit across solid tumors remains inconsistent. Some patients exhibit significant tumor regression and prolonged survival, whereas others fail to respond—even when treated with the same OV platform—highlighting the need to refine patient selection strategies.

This heterogeneity underscores the critical need for predictive biomarkers to identify patients most likely to benefit from OV therapy. A growing body of evidence suggests that tumor mutational burden (TMB), interferon pathway defects, baseline CD8^+^ T cell infiltration, PD-L1 expression, and gut microbiota composition may modulate OV efficacy [22,23,24]. For instance, tumors with impaired antiviral signaling pathways may be more permissive to viral replication, while those with pre-existing immune infiltration may synergize more effectively with OV-induced immunity [25,26]. Despite these insights, most clinical trials to date have not incorporated biomarker-driven stratification, resulting in a “one-size-fits-all” approach that may dilute therapeutic impact.

As the field moves toward precision oncology, there is an urgent need to integrate multi-dimensional biomarker data genomic, immunologic, and microbiome-related into the design and interpretation of OV trials [27,28,29,30]. This becomes especially pertinent with the increasing use of combination therapies, such as pairing OV with immune checkpoint inhibitors (ICIs), which require a deeper understanding of biomarkers that predict synergy, resistance, or toxicity.

Although prior meta-analyses have evaluated the general efficacy and safety of OV therapies, none have comprehensively synthesized the predictive biomarker landscape across cancer types and OV platforms [31,32,33,34,35]. This gap limits the translation of rapidly accumulating biomarker evidence into clinical practice. Therefore, this systematic review and meta-analysis was conducted to address three key objectives: (1) to evaluate the efficacy of OV therapy in solid tumors in terms of objective response rate (ORR), progression-free survival (PFS) and overall survival (OS); (2) to assess the safety profile of OV-based regimens; and (3) to synthesize the available evidence on biomarkers associated with treatment response and resistance. By integrating findings across diverse tumor types and OV modalities, we aim to inform future biomarker-guided strategies for optimizing patient outcomes in oncolytic virotherapy.

## 2. Methods

### 2.1. Study Design and Registration

This systematic review and meta-analysis was conducted in accordance with the Preferred Reporting Items for Systematic Reviews and Meta-Analyses (PRISMA) 2020 guidelines. The study protocol was developed a priori and followed established methodologies for conducting meta-analyses. The protocol is registered with PROSPERO (CRD420251044228).

### 2.2. Eligibility Criteria

We included studies that met the following criteria:

Inclusion Criteria:

- Population: Patients with any form of cancer treated with an oncolytic virus (OV), either as monotherapy or in combination with other treatments.
- Intervention: Administration of a genetically engineered or naturally occurring OV (e.g., T-VEC, adenovirus, reovirus, vaccinia, measles virus).
- Outcomes: Must report at least one of the following:
  ○ ORR;
  ○ PFS;
  ○ OS;
  ○ Biomarker levels.
- Study Types: Randomized controlled trials (Phase II and Phase III).

Exclusion Criteria:

- Observational studies, cohort studies, case reports, reviews, editorials, and conference abstracts.
- Preclinical in vitro or animal studies without human data.
- Studies without sufficient data for extraction or quantitative analysis.

### 2.3. Information Sources and Search Strategy

A comprehensive literature search was conducted in MEDLINE (via PubMed), Embase, Cochrane CENTRAL, ProQuest and Scopus from inception through May 2025. The search strategy combined controlled-vocabulary (MeSH) terms and free-text keywords to capture studies of oncolytic virus therapy in solid tumors, as follows: oncolytic viruses, virotherapy, t-vec, ov, cancer, tumor, neoplasms, biomarkers, objective response rate, progression-free survival, overall survival, etc.

### 2.4. Study Selection

All search results were imported into EndNote and deduplicated. Two independent reviewers screened titles and abstracts. Potentially eligible studies underwent full-text review. Disagreements were resolved through discussion or adjudication by a third reviewer.

### 2.5. Data Extraction

Data were extracted independently by two reviewers using a standardized data collection form. The following variables were recorded: study characteristics: author, year, country, design, sample size; patient characteristics: cancer type, stage; oncolytic virus: type, genetic modifications, route of administration, biomarkers; endpoints: ORR, PFS, OS and their association with biomarker levels; statistical outcomes: odds ratios (ORs), hazard ratios (HRs) and confidence intervals (CI).

### 2.6. Risk of Bias Assessment

The risk of bias for RCTs was assessed using the Cochrane Risk of Bias 2.0 (RoB 2) tool. Publication bias was evaluated using funnel plots and Egger’s test, with visual asymmetry suggesting potential bias.

### 2.7. Data Synthesis and Statistical Analysis

Effect estimates were synthesized using a random-effects model to account for expected clinical and methodological heterogeneity among the included studies [36,37,38]. This heterogeneity encompassed variations in tumor types, oncolytic virus platforms (e.g., T-VEC, adenovirus, reovirus), and treatment modalities (monotherapy versus combination therapy). Results were expressed as HR with 95% CIs for time-to-event outcomes, including PFS and OS, and as OR for categorical outcomes such as ORR. To quantify inter-study variability, the I^2^ statistic was employed, with values of 25%, 50%, and 75% interpreted as indicating low, moderate, and high heterogeneity, respectively. The statistical significance of heterogeneity was assessed using Cochran’s Q test, where a *p*-value below 0.10 was considered suggestive of substantial heterogeneity. To further explore sources of heterogeneity, we conducted pre-specified subgroup analyses stratified by oncolytic virus type (T-VEC vs. non–T-VEC), tumor type (melanoma vs. other solid tumors), and clinical outcomes (ORR, PFS, OS). These analyses aimed to assess potential effect modification and improve the accuracy of summary estimates in light of biological and clinical diversity. Additionally, prediction intervals were calculated for each pooled effect size to estimate the likely range of effects in future similar studies. When applicable, publication bias was assessed using funnel plots. All statistical procedures were executed using SPSS version 29.0.

### 2.8. Ethics and Data Availability

This study utilized published data and did not involve human participants or require ethical approval. All extracted data and analysis code will be made available upon publication in an open-access repository.

## 3. Results

### 3.1. Qualitative Synthesis

#### 3.1.1. Study Characteristics

The studies were selected as per the PRISMA 2020 flow diagram (Supplementary Figure S1). A total of 36 trials (Phases II and III) met the inclusion criteria for this systematic review. The majority (30 trials) were Phase II, while 6 were Phase III RCTs. These studies were published between 1992 and 2023, with the earliest trial in 1992 [39] and the most recent in 2023 [40,41]. Cumulatively, the trials enrolled around 4190 participants across all arms. The trials were geographically diverse, involving sites across North America, Europe, and Asia. Notably, all included trials evaluated solid tumors. The included RCTs spanned a wide range of tumor types. Melanoma was the most common disease focus, with 12 trials in advanced or adjuvant melanoma populations [42,43,44,45,46,47]. Hepatocellular carcinoma (HCC) was the next most common, with 5 trials [48,49,50,51,52]. There were 2 trials in colorectal cancer [39,53] and 2 in non–small cell lung cancer (NSCLC) [54,55]. Two trials in nasopharyngeal carcinoma [56,57] were included, and one trial was conducted in extensive-stage small cell lung cancer [58]. Single trials were identified for metastatic breast cancer [59], pancreatic cancer [60] and ovarian cancer [61]; two trials for prostate cancer [62,63]; and one each for advanced sarcoma [64], glioblastoma [65], head and neck carcinoma [66], cervical cancer [67] and malignant effusions [68], among others.

Most trials were single-center or multi-center national studies, although some later-phase trials were multinational [40,41]. Across the 36 trials, oncolytic virus (OV) platforms included DNA viruses (herpesvirus, adenovirus, vaccinia) and RNA viruses (reovirus, Seneca Valley virus, etc.), administered either as monotherapy or in combination with other treatments (chemotherapy, immune checkpoint blockade, or radiotherapy). In summary, the 36 trials represent a diverse set of Phase II and III studies across at least a dozen cancer types, with sample sizes ranging from as low as 10 patients [48] to nearly 700 patient (Table 1) [40,41].

#### 3.1.2. Quality Assessment

**Figure 1:**
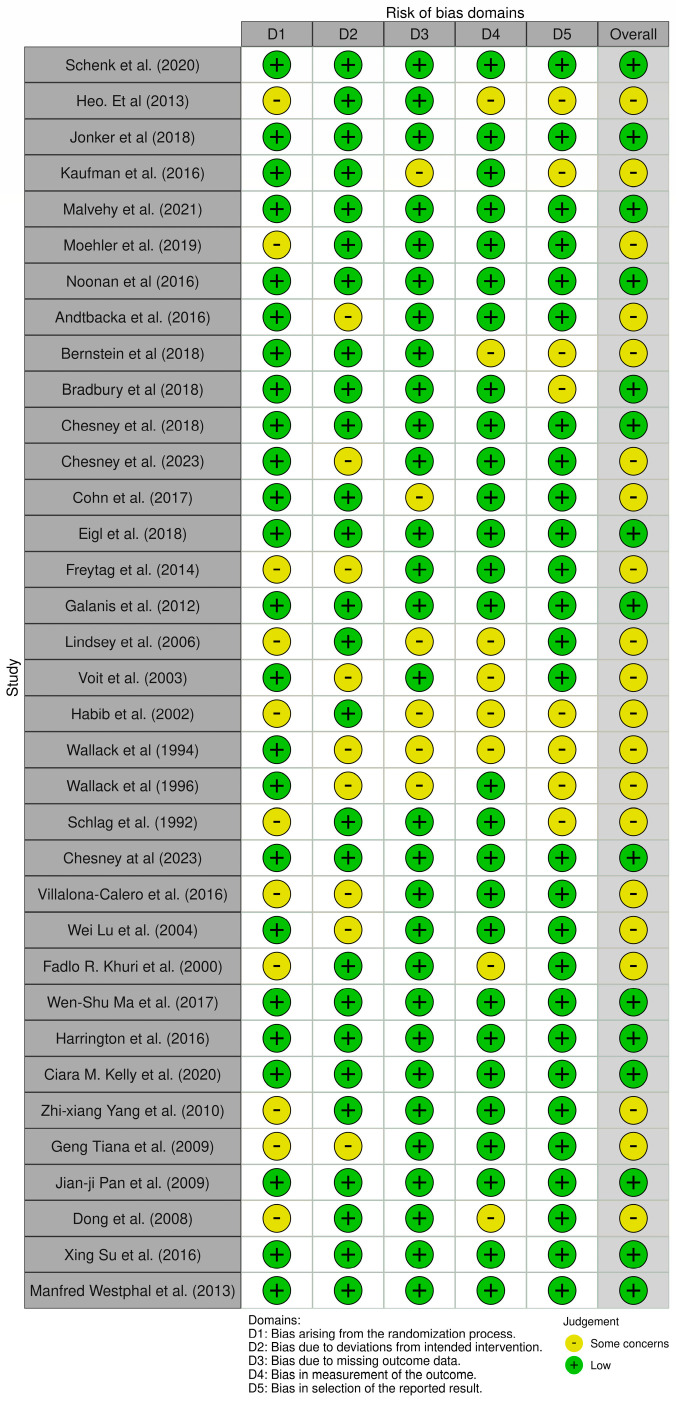
Traffic-light visualization of risk of bias judgments (RoB 2.0) across RCTs included in the review. Created using the ROBVIS tool, a visualization tool for risk-of-bias assessments (https://www.robvis.net/, accessed on 1 May 2025) [39,40,41,42,45,46,47,48,49,50,51,52,53,54,55,56,57,58,59,60,61,62,63,64,65,66,67,68,69,70,71,72,73,74,75].

Risk-of-bias appraisal with the RoB 2.0 framework indicated a generally strong methodological footing for the evidence base. Fifteen of the 36 randomized trials (42%) fulfilled low-risk criteria across all domains. The remaining twenty-one studies (58%) were flagged as having ‘some concerns’, almost invariably because allocation-concealment procedures or prespecified analytic plans were incompletely reported issues that raise the specter of selective reporting but did not materially affect outcome measurement or data completeness, which were largely sound. Notably, no trial reached a high-risk designation in any domain. Collectively, these findings support a moderate-to-high level of confidence in the pooled efficacy estimates, although the domain-specific uncertainties should be borne in mind when interpreting subgroup effects and secondary endpoints (Figure 1).

### 3.2. Efficacy

#### 3.2.1. Melanoma

OV therapy has been most extensively tested in melanoma, with both phase II and III trials. The phase III OPTiM trial (talimogene laherparepvec, T-VEC, an oncolytic HSV, vs. GM-CSF) demonstrated a significantly higher ORR with T-VEC (26% vs. 6% in the control arm) and a trend toward improved OS (HR 0.79, 95% confidence interval 0.62–1.00) [42,43,44]. Although the OS difference in the overall OPTiM population narrowly missed significance (*p* = 0.051) file-(Ref) [43], a pre-specified subgroup analysis of patients with earlier-stage metastatic disease showed a marked survival benefit: in stage IIIB–IVM1a melanoma, median OS was 41.1 months with intralesional T-VEC vs. 21.5 months with GM-CSF (HR 0.57, *p* < 0.001) [75].

A phase III trial combining T-VEC with pembrolizumab (PD-1 inhibitor) in advanced melanoma (Masterkey-265) did not improve PFS or OS compared to pembrolizumab alone (HR for PFS 0.86, *p* = 0.13; HR for OS 0.96, *p* = 0.74) [40,41,45]. The combination did, however, yield a higher response rate (ORR 48.6% vs. 41.3%, with more complete responses) than checkpoint inhibitor [40,41], although this did not translate into a survival advantage. In contrast, a phase II trial of T-VEC combined with ipilimumab (CTLA-4 inhibitor) showed a significantly improved ORR (39% vs. 18%, *p* = 0.002) compared to ipilimumab monotherapy [45], with a nonsignificant trend toward prolonged PFS (median 8.2 vs. 6.4 months, HR 0.83). Notably, single-arm studies of intralesional T-VEC in injectable melanoma reported durable local responses (overall response 28%, including 14% complete response) and regression of non-injected lesions, consistent with a systemic immune-mediated effect.

Beyond T-VEC, earlier-generation virotherapy trials in melanoma had mixed results. An adjuvant vaccinia oncolysate vaccine (VMO) in resected stage II melanoma did not significantly prolong disease-free or OS in the overall population, showing benefit only in retrospective subsets (male subpopulation) [46,47]. Likewise, a viral melanoma vaccine plus high-dose IL-2 immunotherapy yielded only a 12.5% partial response rate (with responses confined to the IL-2–augmented arm). A small phase II trial of Newcastle disease virus (NDV)-modified autologous tumor vaccine in resected stage III melanoma suggested reduced recurrence rates versus matched controls (61% vs. 87% recurrence at follow-up) [73], but survival outcomes were not clearly improved.

Melanoma trials indicate that oncolytic viruses can induce objective tumor responses particularly in injectable dermal metastases and in one case improved survival in a lower disease-burden subgroup. However, OV monotherapy has shown only modest survival benefit overall, and combining OV with systemic immunotherapy has yet to demonstrate a clear OS advantage in advanced melanoma.

#### 3.2.2. Hepatocellular Carcinoma (HCC)

Five phase II trials evaluated oncolytic virotherapy in advanced HCC. A randomized dose-finding study of intravenous oncolytic vaccinia (Pexa-Vec, JX-594) reported a doubling of median survival with a high dose vs. a low dose (14.1 vs. 6.7 months, HR 0.39, *p* = 0.020) [49], suggesting a dose-dependent efficacy signal. In contrast, a larger phase IIb trial (TRAVERSE) of Pexa-Vec versus best supportive care (post-sorafenib) did not demonstrate an OS improvement the study was stopped early for futility, and no significant survival difference was reported [50].

Similarly, two Chinese trials testing intratumoral or intra-arterial adenovirus-p53 (rAd-p53) in HCC showed no statistically significant survival benefit over standard therapy. In one RCT, 1-year OS was 90% with rAd-p53 plus radiotherapy vs. 70% with radiotherapy alone (HR 0.61), but this difference was not significant given the small sample [52]. Another pilot trial of rAd-p53 added to transarterial chemoembolization (TACE) found no improvement in median OS (12.8 vs. 10.4 months, *p* = 0.87) or time to progression [51]. An earlier small trial of an E1B-deleted adenovirus (dl1520) in HCC [48] demonstrated only a 10% partial response rate.

Overall, HCC trials to date have not shown consistent survival gains from virotherapy. While one high-dose vaccinia study suggested prolonged survival, later-phase trials failed to confirm a significant OS benefit. Objective response rates in HCC were generally low (mostly disease stabilization); thus, oncolytic viruses in HCC have shown limited efficacy, and no clear improvement in PFS or OS in randomized settings.

#### 3.2.3. Colorectal and Pancreatic Cancers

Two trials assessed oncolytic virotherapy in colorectal cancer. A modern Phase II trial in metastatic colorectal cancer [53] evaluated systemic oncolytic reovirus (pelareorep) in combination with FOLFOX6-bevacizumab chemotherapy. This trial did not meet its efficacy goals: the pelareorep arm had a shorter median PFS (7 months vs. 9 months in the control arm; HR 1.59, *p* = 0.046) and no improvement in median OS (19.2 vs. 20.1 months; HR 1.22, *p* = 0.38). Interestingly, the virus-combination arm showed a higher objective response rate (53% vs. 35% in the control, OR 2.5), suggesting that pelareorep induced more tumor shrinkage even though this did not translate into longer disease control.

In contrast, an older Phase II adjuvant immunotherapy trial [39] in colorectal cancer patients with resected liver metastases reported a favorable impact on recurrence rates. In that study, an autologous tumor cell vaccine infected with Newcastle disease virus (NDV) was given after surgery, and the vaccinated group had a lower tumor recurrence rate (61% vs. 87% in the matched surgery-alone control group). This suggests a trend toward improved disease-free survival with the NDV-based vaccine, although no significant OS difference was reported. The evidence in colorectal cancer is limited: one RCT showed increased response rates but no survival benefit with oncolytic reovirus therapy, while another indicated a potential reduction in relapse with an oncolytic vaccine approach in the adjuvant setting.

In metastatic pancreatic cancer, a phase II trial [60] added intravenous reovirus to carboplatin/paclitaxel. No efficacy advantage was seen: median PFS was 4.9 months with virus vs. 5.2 months control (*p* = 0.6), and there was no improvement in response rate or OS (outcomes were nearly superimposable between arms).

Taken together, colorectal and pancreatic trials suggest that oncolytic reovirus did not enhance and in the case of colorectal cancer may have slightly reduced the efficacy of standard chemotherapy. These cancers did not exhibit meaningful survival or disease control improvements from OV therapy in phase II testing.

#### 3.2.4. Lung Cancers

In non–small cell lung cancer (NSCLC), two phase II trials evaluated systemic oncolytic therapy in combination with chemotherapy. Neither showed an efficacy advantage. A 166-patient RCT of carboplatin/paclitaxel ± reovirus (intravenous pelareorep) in advanced NSCLC reported no significant differences in outcomes: median OS 7.8 vs. 7.4 months (combination vs. chemo alone; HR 0.98) and median PFS 3.0 vs. 2.8 months (HR 0.90, *p* = 0.53) [54]. Tumor response rates were nearly identical in both arms (14% ORR) [54] indicating no added benefit from the virus.

A biomarker-selected single-arm trial [55] in KRAS-mutant NSCLC reported a 31% objective response rate with pelareorep plus chemotherapy, but without a control group, this serves mainly to show feasibility and some activity. In extensive-stage small cell lung cancer, a Phase II placebo-controlled trial [58] evaluated the systemic oncolytic picornavirus Seneca Valley (NTX-010) as maintenance therapy. This trial was negative for efficacy: there was no significant difference in PFS between the NTX-010 and placebo arms (median PFS 1.7 months in both; HR 1.03, *p* = 0.92). OS was also not improved (HR 1.53, 95% CI 0.76–3.06). Thus, in lung cancers, oncolytic virotherapy has not yet shown a clear clinical benefit in Phase II trials, aside from isolated responses in single-arm settings.

#### 3.2.5. Other Solid Tumors

Evidence in other cancers comes from smaller Phase II trials. In metastatic breast cancer, a randomized Phase II [59] of paclitaxel ± pelareorep showed a non-significant trend toward improved OS with the virus (17.4 vs. 10.4 months; HR 0.65, *p* = 0.10) but no difference in PFS (3.78 vs. 3.38 months) or ORR. In metastatic pancreatic cancer, a Phase II [60] of chemotherapy ± pelareorep was null for efficacy, with no PFS improvement (median 5 months in both arms, *p* = 0.6) and no reported OS benefit. In platinum-refractory ovarian cancer, a Phase IIB trial [61] of weekly paclitaxel ± reovirus showed no advantage: median PFS 4.4 vs. 4.3 months (HR 1.11) and ORR 17.4% vs. 20% (virus vs. control), indicating no efficacy signal.

Two trials in prostate cancer had differing designs: one [63] in intermediate-risk localized prostate cancer found that adding an oncolytic adenovirus-mediated gene therapy to radiation therapy significantly reduced the rate of residual tumor on biopsy (33% vs. 58% positivity two years post-treatment), suggesting improved local disease control. However, in metastatic castration-resistant prostate cancer, an oncolytic reovirus + docetaxel trial [62] showed no efficacy benefit; in fact, the virus arm had a shorter OS (19.1 vs. 21.1 months for control, HR 1.83, *p* = 0.06), indicating the combination was not effective. In advanced sarcoma, a single-arm Phase II [64] of T-VEC plus pembrolizumab reported a 35% ORR, including some durable responses, but no comparator arm was available.

Several RCTs investigated oncolytic virus therapy in combination with standard local treatments. In nasopharyngeal carcinoma (NPC), ref. [57] found that adding a p53 gene therapy adenovirus to radiotherapy did not significantly improve 5-year OS (66.7% vs. 59.2%, *p* = 0.34). Likewise, 5-year PFS was similar between arms.

However, a three-arm trial in recurrent NPC [56] showed a positive signal: the combination of rAd-p53 with chemoradiotherapy achieved a higher tumor response rate and longer PFS compared to chemoradiotherapy alone (the rAd-p53 + CRT arm had the best outcomes, although exact figures were not reported). In locally advanced cervical cancer, a Phase II trial [67] combining rAd-p53 with radiotherapy suggested a trend toward improved survival (5-year OS HR 0.55) and disease control, but these differences were not statistically significant. A unique study in malignant pleural/peritoneal effusions [68] demonstrated that intrapleural rAd-p53 gene therapy alongside cisplatin significantly improved effusion control rates (63.0% vs. 42.9%, *p* < 0.05).

Lastly, an oncolytic virotherapy for high-grade glioma was tested in a Phase III trial [65], using a herpesvirus-based gene therapy vector with prodrug activation). This trial did not show efficacy: time to death or re-intervention was not improved (the composite endpoint favored the control arm; HR for the treatment arm 1.53, 95% CI 1.13–2.07), and there was no OS benefit (HR 1.18). In summary, outside of melanoma and HCC, most tumor-specific trials of oncolytic viruses have reported no significant improvements in traditional efficacy endpoints (PFS/OS). Some notable exceptions include improved local control or response rates in certain settings—for example, the enhanced biopsy-negative rate in prostate cancer with virotherapy [63], and higher effusion resolution rates in the intrapleural adenovirus trial [68]. Additionally, combining oncolytic viruses with other therapies (chemotherapy, radiotherapy, or immunotherapy) has shown additive tumor response in a few studies [42,43,44,56], but these improvements have not consistently translated into longer.

### 3.3. Safety

The overall oncolytic virus therapy was found to have an acceptable safety profile across diverse platforms and tumor types. This favorable safety profile is underpinned by multiple mechanistic factors: oncolytic viruses are genetically engineered or naturally attenuated to replicate preferentially in malignant cells (e.g., via deletions of virulence genes or use of tumor-specific promoters), while sparing normal tissues. Furthermore, the host innate immune system rapidly recognizes and clears viral particles from non-tumor sites, limiting systemic biodistribution and toxicity. Intratumoral administration of many agents further confines replication to the tumor microenvironment, reducing off-target effects. The most common adverse events (AEs) were mild to moderate flu-like symptoms—fever, chills, fatigue, and nausea were frequently observed with viral therapies (e.g., reported in trials of vaccinia virus and reovirus by [49,60]—as well as injection-site reactions for intratumoral agents [69]. These AEs were generally grade 1–2 in severity and self-limited. For instance, intralesional T-VEC in melanoma was associated with transient local inflammation and systemic flu-like symptoms in most patients, but very few grade ≥3 events [42,44,69].

Similarly, intravenous oncolytic reovirus led to expected flu-like symptoms and cytopenias (when combined with chemotherapy) but no significant increase in serious toxicity compared to chemotherapy alone [54,60]. Severe toxicities were infrequent across these studies. Grade 3–4 adverse events tended to reflect either the background therapy (e.g., myelosuppression from co-administered chemotherapies) or underlying disease complications, rather than direct toxic effects of the viruses. For example, in [59], the rate of severe neutropenia was similar between the pelareorep + paclitaxel arm and chemotherapy-alone arm, attributable to paclitaxel in both groups. Several trials specifically monitored for dose-limiting toxicities: [63] examined acute toxicity ≤ 90 days in a prostate cancer virotherapy trial and reported no significant increase in high-grade toxicities with the oncolytic adenovirus (acute side effects were comparable to radiation alone).

Likewise, Chesney et al. (2018) [45] noted that adding T-VEC to ipilimumab did not appreciably worsen immune-related adverse events beyond those expected with ipilimumab. Some platform-specific toxicities were reported anecdotally—for instance, high-dose vaccinia virus in HCC was associated with transient fever and hypotension requiring observation [49], and oncolytic HSV-1 (T-VEC) occasionally caused low-grade herpetic lesions at injection sites—but these were manageable and self-resolving, reflecting the balance between targeted viral replication and host antiviral defenses. No trials reported any viral shedding or transmission issues of clinical concern, and laboratory markers (such as neutralizing antibody formation, monitored in several studies) indicated an expected immune response without safety alarm signals (Schenk et al., 2020 [58] reported robust antibody formation but no toxicity correlation). Importantly, treatment-related mortality was rare to nonexistent in these studies. No deaths were attributed to the oncolytic viruses themselves in any of the trials. In the large Phase III melanoma study of T-VEC [42,44], there were no significant differences in treatment-related death between the T-VEC and control arms (no patient deaths were deemed related to T-VEC). Similarly, Chesney et al. (2023) [40,41] reported no treatment-related fatalities in the T-VEC plus pembrolizumab trial, and Schenk et al. (2020) [58] noted no deaths attributable to Seneca Valley Virus versus placebo. When deaths occurred during trials, they were uniformly caused by disease progression or complications of standard therapies rather than the experimental virus. For example, in Noonan et al. (2016) [60] (metastatic pancreas study), several patients died from advanced disease, but none of those were considered related to reovirus.

Overall, oncolytic virotherapy did not appear to add significant toxicity burden. The therapy was generally well tolerated with a safety profile consisting mostly of mild flu-like symptoms and injection-site reactions (across diverse viruses and tumor types), and with no consistent pattern of serious adverse events beyond those associated with conventional treatments. Mechanistically, the combination of tumor-selective viral replication, engineered attenuation, localized delivery, and rapid innate immune clearance explains why even systemically administered oncolytic viruses remain so well tolerated. This favorable safety outcome is an encouraging aspect of oncolytic virus therapy noted throughout the literature, reinforcing that while efficacy has varied, the risk profile has remained acceptable in Phase II–III trials [70,71,72,74,76].

### 3.4. Qualitative Synthesis of Biomarker Trends in Oncolytic Virus (OV) Therapy

**Figure 2:**
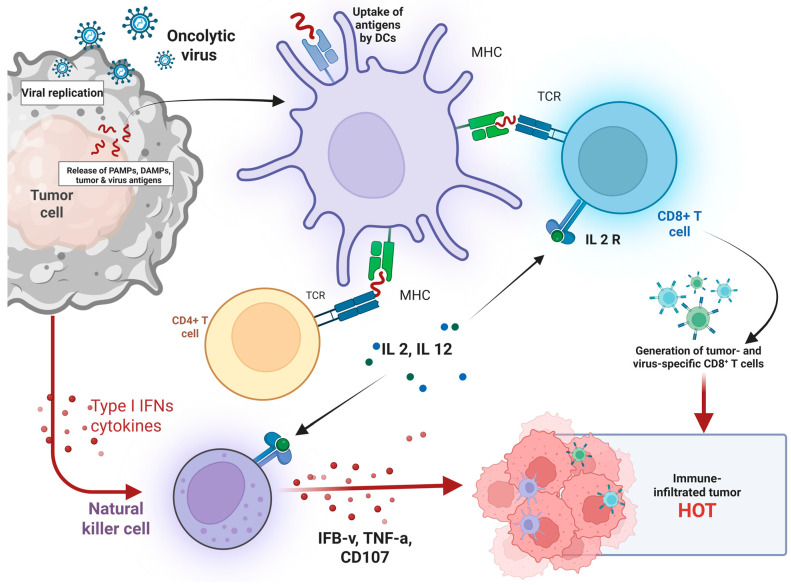
Mechanism of Oncolytic Virus-Induced Antitumor Immunity and Tumor Microenvironment Modulation. Illustrates the immunological cascade initiated by oncolytic virus (OV) therapy. Upon infection of tumor cells, OV replicate and induce tumor lysis, leading to the release of pathogen-associated molecular patterns (PAMPs), damage-associated molecular patterns (DAMPs), and tumor-associated antigens. These antigens are taken up by dendritic cells (DCs), which present them via MHC molecules to CD4^+^ and CD8^+^ T cells, promoting T-cell activation. Activated CD4^+^ T cells secrete interleukins (IL-2, IL-12), which further stimulate cytotoxic CD8^+^ T cells. Natural killer (NK) cells are also activated by Type I interferons and cytokines, contributing to antitumor immunity by releasing IFN-γ, TNF-α, and CD107. Collectively, these responses result in the generation of tumor- and virus-specific CD8^+^ T cells, transforming the tumor microenvironment into a T cell–infiltrated, immunologically active “hot” tumor conducive to immune-mediated tumor clearance. Created in BioRender. Babiker, R. (2025). https://BioRender.com/4561u2v, accessed on 1 May 2025.

The reviewed studies collectively demonstrate that oncolytic virus (OV) therapies, both as standalone agents and in combination with immunotherapy, elicit meaningful biological changes across a range of immunologic, molecular, and surrogate biomarkers. These changes support the immunomodulatory, pro-apoptotic, and systemic effects attributed to OV therapy (Table 2, Figure 2).

### 3.5. Immune Activation in the Tumor Microenvironment

**Figure 3:**
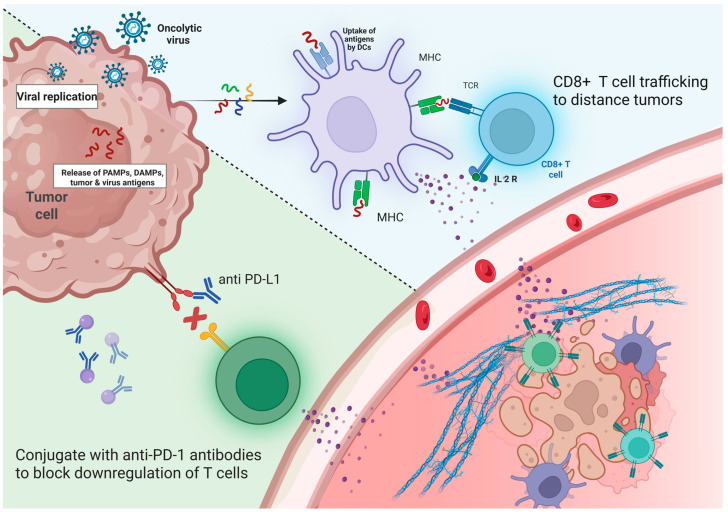
Synergistic Antitumor Mechanism of Oncolytic Viruses and Immune Checkpoint Inhibitors (ICIs). Depicts the combined therapeutic strategy of oncolytic viruses (OV) and immune checkpoint inhibitors (ICIs) in enhancing antitumor immunity. Oncolytic viruses selectively infect and replicate within tumor cells, leading to cell lysis and the release of tumor antigens, pathogen-associated molecular patterns (PAMPs), and damage-associated molecular patterns (DAMPs). These antigens are captured and processed by dendritic cells (DCs), which present them via MHC molecules to CD8^+^ T cells, activating them and inducing the expression of IL-2 receptors. The presence of IL-2 promotes CD8^+^ T cell proliferation and trafficking to distant tumor sites. Concurrently, ICIs such as anti-PD-L1 antibodies block the PD-1/PD-L1 pathway, preventing T cell exhaustion and restoring T cell cytotoxic function. The combined approach transforms the tumor microenvironment into a more immunogenic state, enhancing immune infiltration and improving tumor clearance by reactivating and directing T cells to both primary and metastatic tumor sites. Created in BioRender. Babiker, R. (2025). https://BioRender.com/iaefvgm, accessed on 1 May 2025.

A consistent hallmark of OV therapy, particularly when combined with checkpoint inhibitors, is the increased infiltration of CD8^+^ cytotoxic T cells, along with elevated PD-L1 expression and IFN-γ gene expression in the tumor microenvironment. These findings, reported by Ribas et al. (2017), Shoushtari et al. (2023), and Schwarze et al. (2022) [77,78,79], suggest that OV-induced inflammation can amplify antitumor immunity, especially when paired with immune checkpoint blockade (Figure 3). These biomarkers reflect a robust local and systemic immune activation, which is often associated with favorable clinical responses.

### 3.6. Viral Replication and Host Immune Recognition

Studies by Schenk et al. (2020) and Garcia et al. (2018) [58,80] observed active viral replication and the generation of neutralizing antibodies, indicating successful delivery and recognition of the viral vector by the host immune system. While these viral biomarkers consistently increased during treatment, their direct clinical relevance remains uncertain, highlighting a need for further validation in larger cohorts.

### 3.7. Systemic Immune Modulation

Peripheral immune markers also shifted favorably in response to OV therapy. Noonan et al. (2016) and Mahalingam et al. (2020) [60,81] reported decreases in immunosuppressive CTLA4^+^ regulatory T cells and increases in cytotoxic and memory T-cell subsets. These systemic changes support the view that OV can extend their immunomodulatory effects beyond the injection site, potentially contributing to abscopal responses in non-injected lesions.

### 3.8. Molecular Markers of Tumor Suppression

Several studies using recombinant adenoviruses, such as those by Yang et al. (2010), Tian et al. (2009), and Pan et al. (2009) [51,52,57], demonstrated upregulation of pro-apoptotic genes (p53, p21, Bax) and downregulation of angiogenic markers like VEGF. These molecular changes reflect the direct tumor-killing potential of genetically engineered viruses and support the idea of OV therapy as a dual mechanism agent—inducing both immunogenic and apoptotic tumor cell death.

### 3.9. Tumor Burden and Lesion-Level Response

Clinical surrogates like tumor size reduction and systemic lesion-level responses, especially in uninjected tumors, were reported by Andtbacka et al. (2016) and Stahlie et al. (2021) [42,82]. These outcomes were more pronounced in patients with smaller baseline tumor burden, suggesting the potential for patient stratification based on tumor volume.

### 3.10. Emerging Surrogate Clinical Biomarkers

In breast and pancreatic cancer studies, circulating tumor cells (CTCs) and residual cancer burden (RCB) declined following OV therapy, as observed by Bernstein et al. (2018) and Soliman et al. (2020) [59,83]. While these changes were not uniformly predictive, they were generally aligned with imaging-based tumor responses, indicating a possible future role for these non-invasive markers in monitoring therapeutic efficacy.

### 3.11. Quantitative Synthesis

#### Progression-Free Survival

**Figure 4:**
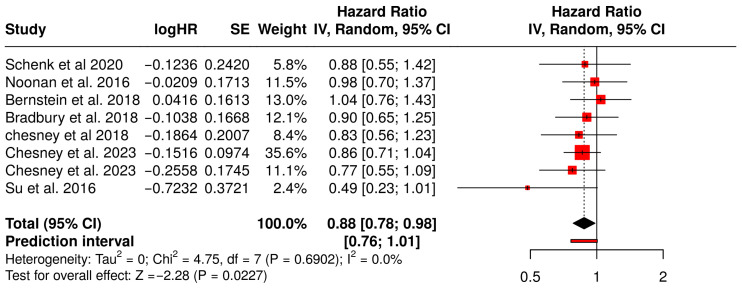
Forest Plot of Progression-Free Survival (PFS) Between Combination Therapy with Oncolytic virus and Monotherapy Groups [40,41,45,54,58,59,60,67].

This meta-analysis explored the impact of combining oncolytic virus therapy with standard medications on PFS in patients diagnosed with various tumors (Figure 4). A total of 8 studies involving 1541 patients were evaluated. PFS is a critical endpoint in oncology research, reflecting the length of time during and after treatment that a patient lives with the disease without it worsening. The pooled hazard ratio (HR) across the studies was 0.88, with a 95% confidence interval ranging from 0.78 to 0.98. This indicates an 12% statistically significant reduction in the risk of disease progression or death in the group receiving the combination therapy compared to those on medications alone.

The relatively narrow confidence interval and the statistical significance (*p* < 0.05) suggest that the observed benefit is likely not due to random chance. Moreover, the analysis did not reveal significant heterogeneity across the studies, suggesting that the effect was consistently observed in different tumor types, populations, and treatment settings. These results collectively imply that the addition of oncolytic viruses to standard treatments provides a meaningful clinical advantage in delaying disease progression, with a consistent treatment effect across multiple independent studies.

Among the studies Chesney et al. (2018) showed a favorable HR of 0.83 (95% CI: 0.56–1.23) in melanoma patients, supporting the efficacy of combination therapy in immunogenic tumors [45]. Bradbury et al. (2018), investigating non-small cell lung cancer (NSCLC), reported an HR of 0.90 (95% CI: 0.65–1.25), consistent with a modest PFS benefit [54]. Although Schenk et al. (2020) [58] and Bernstein et al. (2018) [59] reported HR around 1.03 and 1.04, respectively, indicating minimal differences, the direction of effect remained consistent with the overall analysis. The slight variability in effect sizes across tumor types (e.g., pancreatic, breast, lung cancer) contributed to the robustness of the pooled estimate, with no significant heterogeneity detected.

**Figure 5:**
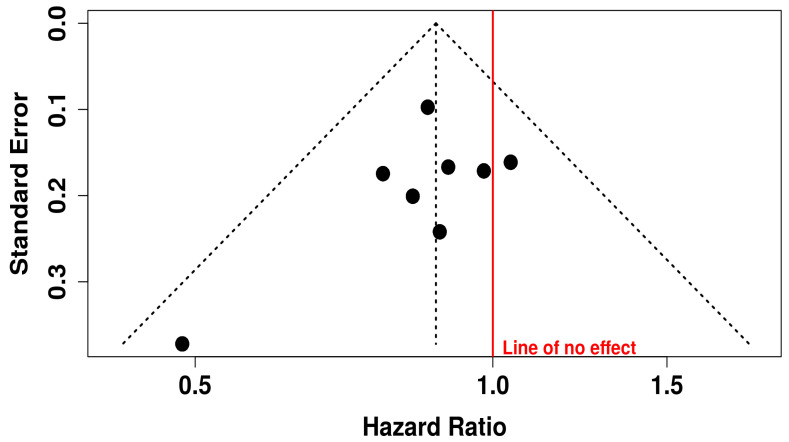
Funnel Plot for Publication Bias in Progression-free Survival (PFS) Studies.

The corresponding funnel plot for PFS does not show marked asymmetry, which implies the absence of major publication bias. This is crucial for interpreting the validity of meta-analytic findings, as publication bias can inflate effect estimates. Egger’s regression test also supports this interpretation, with an intercept of −0.91 (95% CI: −2.6 to 0.78), a t-value of −1.06, and a non-significant *p*-value of 0.33. These findings suggest that the evidence supporting improved PFS with oncolytic virus combination therapy is robust, reliable, and unlikely to be skewed by selective reporting or small-study effects (Figure 5).

### 3.12. Sub-Group Analysis of PFS

#### 3.12.1. PFS in Melanoma Patients Treated with T-VEC

This forest plot presents a subgroup meta-analysis of PFS in melanoma patients treated with Talimogene laherparepvec (T-VEC) across three randomized trials. The pooled hazard ratio was 0.84 (95% CI: 0.72–0.98; *p* = 0.0231), indicating a 16% statistically significant reduction in the risk of disease progression or death with T-VEC therapy compared to control.

The analysis showed no heterogeneity (I^2^ = 0%, *p* = 0.8720), suggesting consistent treatment effects across the included studies. The prediction interval [0.60–1.17] further supports the reliability of the estimated benefit while acknowledging that future studies might observe a range of outcomes depending on trial context.

**Figure 6:**
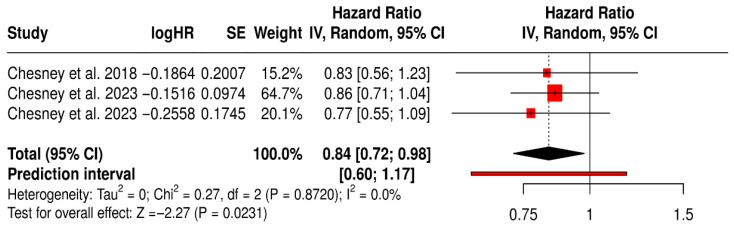
Forest Plot of Progression-Free Survival (PFS) in Melanoma Patients Treated with T-VEC: Subgroup Meta-Analysis of Randomized Trials [40,41,45].

These results reinforce the role of T-VEC in delaying disease progression in advanced melanoma, particularly in patients with accessible injectable lesions and in immunotherapy-sensitive settings (Figure 6).

#### 3.12.2. PFS in Non-Melanoma Solid Tumors Patients with Non–T-VEC Oncolytic Virus

This forest plot presents a subgroup meta-analysis of PFS from six randomized trials evaluating non–T-VEC oncolytic viruses (e.g., reovirus, adenovirus) in various non-melanoma solid tumors, including breast, pancreatic, lung, and ovarian cancers.

**Figure 7:**
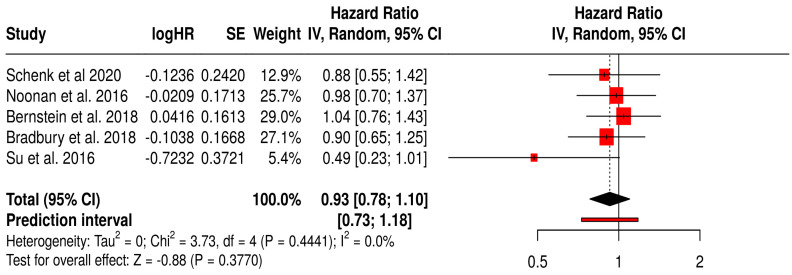
Forest Plot of Progression-Free Survival (PFS) from Subgroup Meta-Analysis of Non–T-VEC Oncolytic Virus Therapies in Non-Melanoma Solid Tumors [54,58,59,60,67].

The pooled hazard ratio was 0.93 (95% CI: 0.78–1.18; *p* = 0.377), indicating no statistically significant improvement in PFS with oncolytic virus therapy compared to standard treatments. The prediction interval [0.73–1.18] suggests consistency in effect across future studies within this clinical context. Importantly, the analysis showed no heterogeneity (I^2^ = 0%, *p* = 0.441), indicating consistent outcomes across included trials. Despite this consistency, the lack of a significant pooled effect highlights the limited efficacy of non-T-VEC OV therapies in delaying disease progression across these diverse cancer types. These findings reinforce the need for targeted, tumor-specific virotherapies and biomarker-driven patient selection to improve outcomes beyond melanoma (Figure 7).

#### 3.12.3. PFS in Solid Tumors Patients with Treated Primarily with Oncolytic Reovirus (Pelareorep)

**Figure 8:**
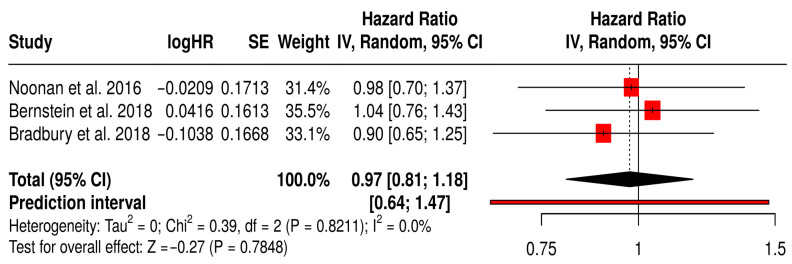
Forest Plot of Progression-Free Survival (PFS) from Subgroup Meta-Analysis of Oncolytic Reovirus Virus Therapies in Solid Tumors [54,59,60].

This forest plot (Figure 8) presents a subgroup meta-analysis of three randomized Phase II trials (Noonan et al., 2016 [60], Bernstein et al., 2018 [59], Bradbury et al., 2018 [54]) that investigated reovirus (Pelareorep) in combination with chemotherapy across diverse solid tumors, including pancreatic, breast, and lung cancers. The pooled hazard ratio for PFS was 0.97 (95% CI: 0.81–1.18; *p* = 0.7848), suggesting that reovirus therapy achieved comparable disease control to standard regimens. While the pooled effect did not cross the threshold for statistical significance, the results demonstrate clinical equipoise between reovirus-based combinations and established chemotherapy. Notably, the prediction interval [0.64–1.47] indicates that future trials are likely to reproduce these consistent outcomes. Importantly, heterogeneity was absent (I^2^ = 0%, *p* = 0.8211), underscoring the reproducibility of reovirus activity across different tumor types and treatment settings. This uniformity suggests a stable therapeutic effect, which is encouraging given the biological diversity of the included cancers.

### 3.13. Overall Survival

**Figure 9:**
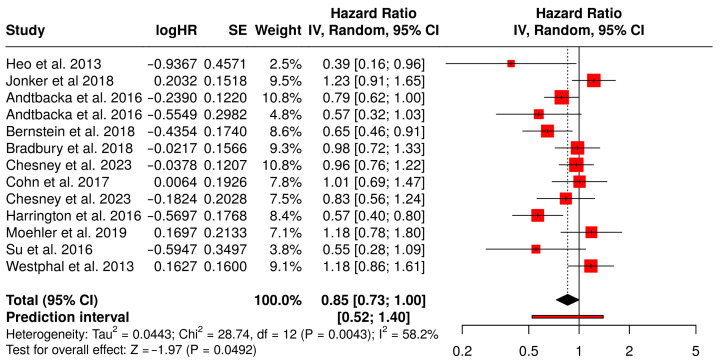
Forest Plot of Overall Survival (OS) Between Combination Therapy with Oncolytic virus and Monotherapy Groups [40,41,42,43,49,50,53,54,59,61,65,67,75].

The meta-analysis of OS involved 13 studies comprising 2598 patients that compared treatment with oncolytic virus therapy plus medications to medications alone in cancer patients (Figure 9). OS is one of the most definitive endpoints in oncology, indicating the length of time from the start of treatment until death from any cause. The pooled hazard ratio (HR) was 0.85 with a 95% confidence interval of 0.73 to 1.00, translating to a 15% statistically significant reduction in the risk of death in patients treated with the combination therapy compared to standard medications alone.

Among the contributing studies, Heo et al. (2013) demonstrated a notable survival benefit in liver cancer patients with an HR of 0.39 (95% CI: 0.16–0.96), suggesting substantial potential for oncolytic virotherapy in hepatocellular carcinoma [49]. Bernstein et al. (2018) also reported a strong effect in metastatic breast cancer patients (HR = 0.65, 95% CI: 0.46–0.91), reinforcing the benefit across solid tumors [59]. In contrast, Jonker et al. (2018), who evaluated metastatic colorectal cancer, showed a slightly adverse but statistically non-significant HR of 1.23 (95% CI: 0.91–1.65), introducing some heterogeneity into the analysis [53]. Andtbacka et al. (2016) contributed data from two cohorts with HRs of 0.79 and 0.57, both favoring combination therapy in melanoma patients [42,43]. This result supports the potential of oncolytic viruses to enhance the efficacy of cancer treatments by improving long-term survival outcomes.

However, the analysis also indicated moderate heterogeneity (I^2^ = 58%), which reflects variability in outcomes among the included studies. This heterogeneity may be attributed to differences in study populations, types of tumors treated, follow-up periods, or the specific oncolytic viruses and drug regimens used. Despite these variations, the statistically significant pooled effect suggests that the survival benefit is a generalizable finding, though it may vary in magnitude depending on clinical context.

**Figure 10:**
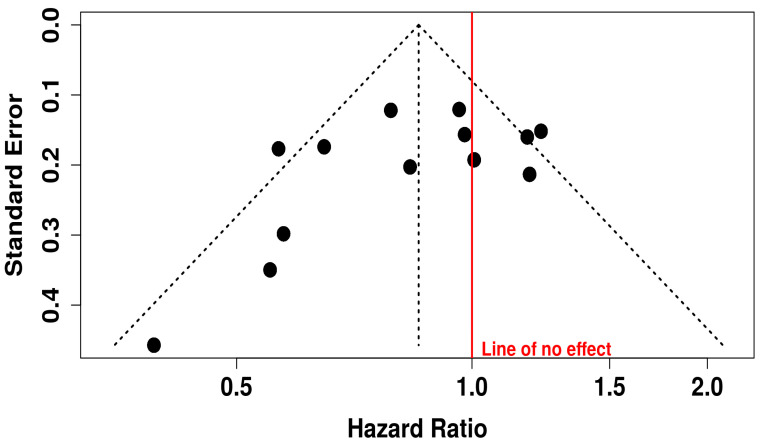
Funnel Plot for Publication Bias in OS Studies.

The funnel plot associated with OS outcomes appears visually symmetrical, indicating no strong evidence of publication bias. This interpretation is further supported by Egger’s test, which yielded an intercept of −2.05 with a 95% CI of −4.59 to 0.48, a t-value of −1.585, and a *p*-value of 0.141. These findings indicate that the likelihood of bias due to selective publication of positive results is low. Thus, the evidence pointing to a survival benefit from the combination therapy appears to be both statistically and clinically meaningful, and largely free from distortion due to small-study effects or publication bias (Figure 10).

### 3.14. Subgroup Analysis of OS

#### 3.14.1. OS in Melanoma Patients Treated with T-VEC

This subgroup meta-analysis evaluated the impact of Talimogene laherparepvec (T-VEC) on OS in melanoma patients across five randomized controlled trials. The pooled hazard ratio was 0.77 (95% CI: 0.63–0.94; *p* = 0.0090), indicating a 23% reduction in the risk of death for patients treated with T-VEC compared to controls.

The prediction interval [0.46–1.27] suggests that while most future studies may observe a survival benefit, the magnitude may vary. Moderate heterogeneity was observed (I^2^ = 46.5%, *p* = 0.113), reflecting some variability in patient populations, trial phases, and combination therapies, yet not undermining the reliability of the pooled estimate.

**Figure 11:**
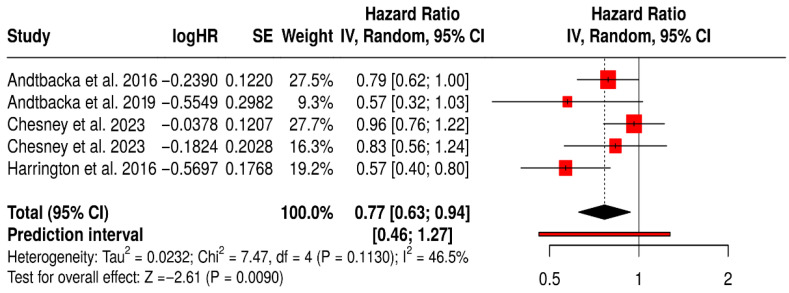
Forest Plot of Overall Survival (OS) in Melanoma Patients Treated with T-VEC: Subgroup Meta-Analysis of Randomized Trials [40,41,42,43,75].

Collectively, these results support the survival benefit of T-VEC in melanoma, especially in settings with accessible injectable lesions and immunogenic tumor microenvironments. The findings also underscore the importance of continued investigation into patient selection and optimal combination strategies to enhance T-VEC efficacy (Figure 11).

#### 3.14.2. OS in Non-Melanoma Solid Tumors Patients Treated with Non-T-VEC

This forest plot summarizes a subgroup meta-analysis of OS from randomized trials using non–T-VEC oncolytic viruses (e.g., reovirus, adenovirus, vaccinia) across various non-melanoma solid tumors, including colorectal, breast, lung, and hepatocellular cancers.

**Figure 12:**
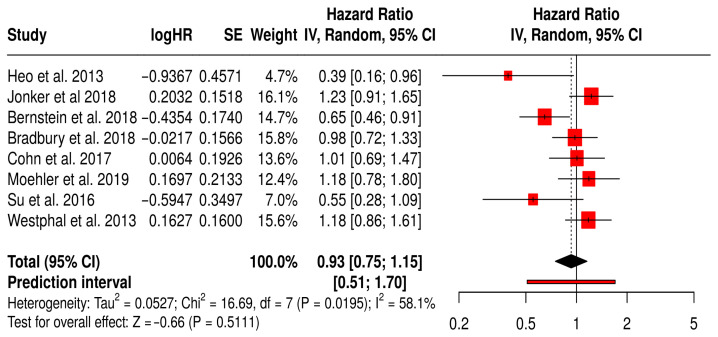
Forest Plot of Overall Survival (OS) from Subgroup Meta-Analysis of Non–T-VEC Oncolytic Virus Therapies in Non-Melanoma Solid Tumors [49,50,53,54,59,61,65,67].

The pooled hazard ratio was 0.93 (95% CI: 0.75–1.15; *p* = 0.2556), suggesting 7% reduction in mortality with oncolytic virus therapy compared to standard treatments, although it was not significant. The wide prediction interval [0.51–1.70] reflects substantial uncertainty about the true effect in future studies and the heterogeneity in clinical contexts. Statistical heterogeneity was moderate (I^2^ = 58%), likely due to variation in cancer types, OV platforms, and combination regimens. Notably, a few individual studies (e.g., Heo et al., 2013 [49], Bernstein et al., 2018 [59]) reported significant OS benefit, whereas others showed no difference or favored the control. These findings indicate that while some non–T-VEC virotherapies may confer survival advantages in select settings, the evidence remains inconsistent across tumor types and platforms. This underscores the need for tumor-specific, biomarker-driven trials to better define which patients benefit most (Figure 12).

#### 3.14.3. OS in Solid Tumors Patients with Treated Primarily with Oncolytic Reovirus (Pelareorep)

**Figure 13:**
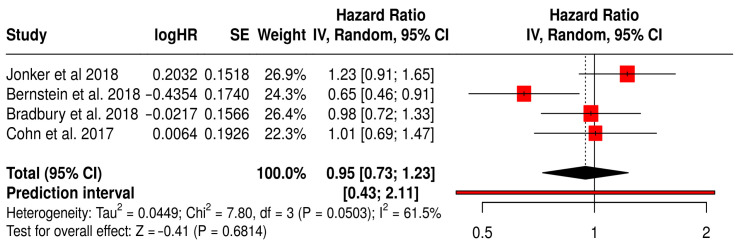
Forest Plot of Overall survival (OS) from Subgroup Meta-Analysis of Oncolytic Reovirus Virus Therapies in Solid Tumors [53,54,59,61].

This forest plot (Figure 13) presents the findings of a subgroup meta-analysis involving four randomized Phase II trials—Jonker et al. (2018) [53], Bernstein et al. (2018) [59], Bradbury et al. (2018) [54], and Cohn et al. (2017) [61]. These studies assessed the efficacy of reovirus (Pelareorep) combined with standard chemotherapy in patients with various solid tumors, including colorectal, breast, lung, and ovarian cancers. The combined hazard ratio for overall survival (OS) was 0.95 (95% CI: 0.73–1.23; *p* = 0.6814), indicating that the addition of reovirus to conventional therapy produced similar survival outcomes. Although the result did not reach statistical significance, the direction of the effect suggests a potential survival benefit for some patients.

The prediction interval (0.43–2.11) highlights the variability in treatment effects, which is expected given the differences in tumor types and study protocols. Nevertheless, the interval still includes the possibility of a clinically meaningful benefit. Moderate heterogeneity was observed (I^2^ = 61.5%, *p* = 0.0503), reflecting the diverse nature of the patient populations and treatments analyzed. Notably, Bernstein et al. (2018) [59] reported a significant OS benefit in patients with metastatic breast cancer (HR 0.65, 95% CI: 0.46–0.91), suggesting that specific tumor subgroups may be more responsive to reovirus-based therapy.

#### Objective Response Rate (ORR)

**Figure 14:**
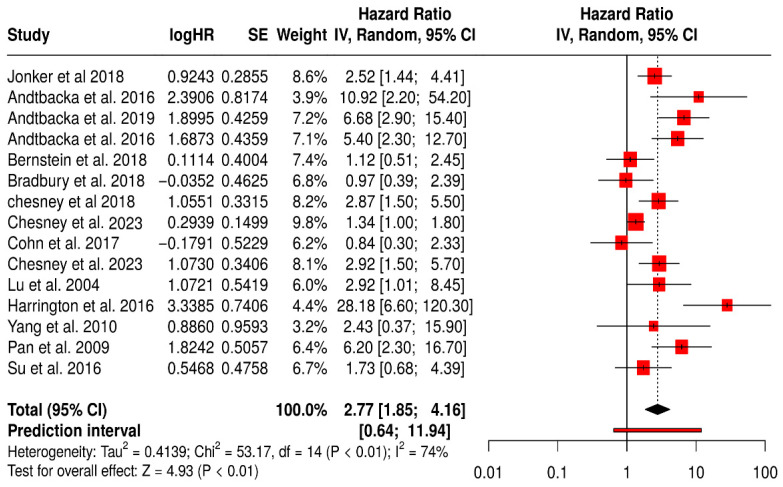
Forest Plot of Objective Response Rate (ORR) Between Combination Therapy with Oncolytic virus and Monotherapy Groups [40,41,42,43,44,45,52,53,54,57,59,61,67,74,75].

This meta-analysis evaluated the ORR across 15 studies involving a total of 3009 patients comparing those receiving a combination of oncolytic virus therapy and medications versus those receiving medications alone (Figure 14). The objective response rate is a direct measure of a treatment’s anti-tumor activity, reflecting the proportion of patients whose tumors shrink by a predefined amount. The pooled hazard ratio was 2.77, with a 95% confidence interval of 1.85 to 4.16, and a highly significant *p*-value (<0.01). This result indicates that the addition of oncolytic virus therapy nearly triples the odds of achieving a clinical response to treatment compared to standard therapy alone.

This magnitude of benefit is both statistically robust and clinically significant, suggesting that oncolytic virus therapy could substantially improve tumor regression in patients. Among the most impactful studies, Andtbacka et al. (2016) reported extremely high ORs of 5.4 and 10.9 in melanoma cohorts, while the 2019 follow-up by the same author further reinforced these findings with an OR of 6.7 (95% CI: 2.9–15.4) [42,43]. These dramatic improvements in ORR underscore the potent immunogenic effects of oncolytic viruses in melanoma. Jonker et al. (2018) [53], working with colorectal cancer patients, reported a positive OR of 2.52 (95% CI: 1.44–4.41), whereas Bernstein et al. (2018) in metastatic breast cancer showed a modest and non-significant benefit (OR = 1.11, 95% CI: 0.51–2.45) [59].

Nevertheless, the heterogeneity across studies was considerable, as reflected by an I^2^ value of 74%. This suggests meaningful variability in the results, which may arise from differences in patient characteristics, tumor biology, treatment protocols, or assessment methods. The wide prediction interval (0.64 to 11.94) also implies that the effectiveness of the combination therapy may vary significantly in future studies or different clinical settings.

**Figure 15:**
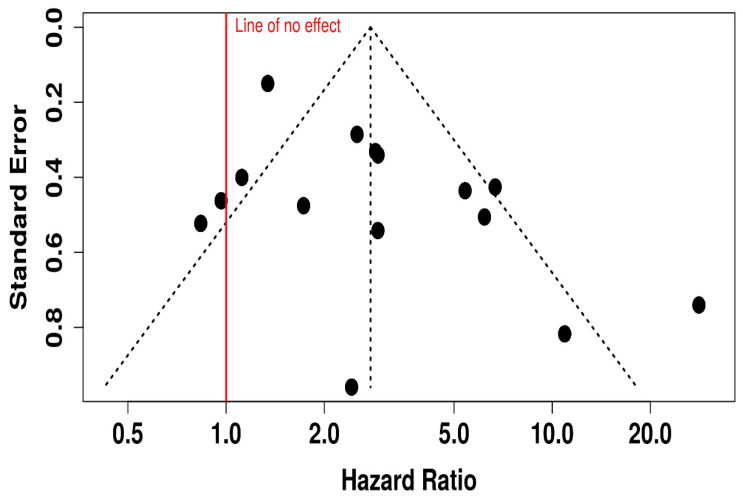
Funnel Plot for Publication Bias in Objective Response Rate (ORR) Studies.

This funnel plot evaluates potential publication bias in studies assessing ORR for oncolytic virus plus medication versus medication alone (Figure 15). While the majority of data points cluster symmetrically around the central vertical axis (indicating the overall effect), there appears to be some asymmetry, particularly on the right-hand side with several studies showing large HR (HR > 5), suggesting possible small-study effects or selective publication of positive findings. Additionally, fewer studies are seen on the lower left side of the plot, which might indicate that studies showing lower or null effects were less likely to be published or detected. This asymmetry, especially with such a wide spread in HR and standard errors, raises concerns about potential publication bias or heterogeneity.

### 3.15. Subgroup Analysis of ORR

#### 3.15.1. ORR in Melanoma Patients Treated with T-VEC

The forest plot for the T-VEC–melanoma subgroup shows a statistically significant improvement in ORR with T-VEC therapy across included studies. The pooled OR was 4.50 (95% CI: 2.22–9.14; *p* < 0.0001), indicating that patients treated with T-VEC were over four times more likely to achieve an objective response compared to controls.

However, substantial heterogeneity was observed (I^2^ = 84.6%, *p* < 0.0001), reflecting variability in study design, patient populations, and treatment settings. The wide prediction interval also suggests that future studies may observe a broad range of effect sizes. Despite this heterogeneity, all individual studies favored T-VEC, supporting its robust antitumor activity in melanoma.

**Figure 16:**
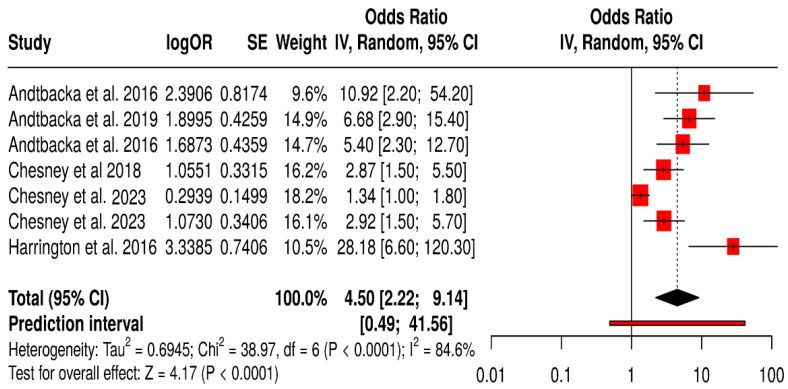
Forest Plot of Objective Response Rate (ORR) in Melanoma Patients Treated with T-VEC Across Randomized Controlled Trials [40,41,42,43,44,45,75].

These findings highlight the consistent efficacy of T-VEC in inducing tumor regression in melanoma patients, especially when used intralesionally in immunogenic tumors. Nonetheless, the observed heterogeneity underscores the importance of context-specific factors such as disease stage, lesion accessibility, and combination strategies (Figure 16).

#### 3.15.2. ORR in Non-Melanoma Solid Tumors Patients Treated with Non-T-VEC

This forest plot presents a subgroup meta-analysis of ORR from randomized trials evaluating non–T-VEC oncolytic virus (OV) therapies across non-melanoma solid tumors. The pooled ORR is 1.86 [95% CI: 1.18–2.94], indicating a statistically significant improvement in tumor response with OV therapy compared to standard treatments (Z = 2.66, *p* = 0.0078). This suggests that patients treated with other oncolytic viruses (e.g., reovirus, adenovirus) in cancers such as colorectal, lung, ovarian, breast, or cervical cancers were nearly twice as likely to exhibit an objective response.

**Figure 17:**
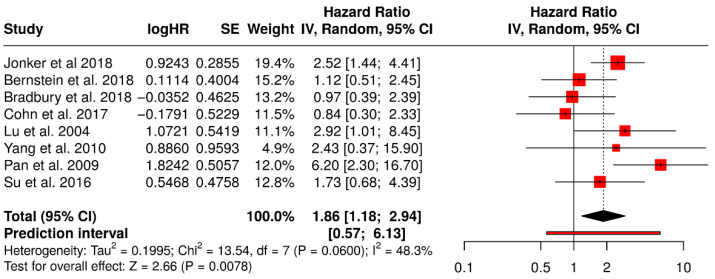
Forest Plot of Objective Response Rates (ORR) from Subgroup Meta-Analysis of Non–T-VEC Oncolytic Virus Therapies in Non-Melanoma Solid Tumors [52,53,54,57,59,61,67,74].

The heterogeneity was moderate (I^2^ = 48.3%, *p* = 0.060), reflecting variability in tumor types, viral platforms, and treatment combinations but not at a level that undermines the validity of the pooled estimate. The prediction interval [0.57–6.13] indicates that while most future studies may still find a beneficial effect, variability in effect size should be expected. Collectively, this analysis supports the broader applicability of OV therapy beyond melanoma, though with generally smaller and more variable effects compared to T-VEC in melanoma (Figure 17).

#### 3.15.3. ORR in Solid Tumors Patients with Treated Primarily with Oncolytic Reovirus (Pelareorep)

**Figure 18:**
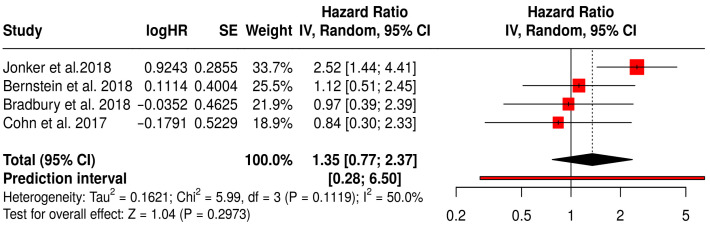
Forest Plot of ORR (ORR) from Subgroup Meta-Analysis of Oncolytic Reovirus Virus Therapies in Solid Tumors [53,54,59,61].

This forest plot (Figure 18) presents a subgroup meta-analysis of four randomized Phase II trials—Jonker et al. (2018) [53], Bernstein et al. (2018) [59], Bradbury et al. (2018) [54], and Cohn et al. (2017) [61]—that investigated the use of reovirus (Pelareorep) in combination with chemotherapy across a range of solid tumors. The pooled hazard ratio for objective response rate (ORR) was 1.35 (95% CI: 0.77–2.37; *p* = 0.2973), indicating a non-significant trend toward improved response rates in patients receiving reovirus-based therapy compared to chemotherapy alone. Although not statistically significant, the point estimate suggests a potential advantage in tumor reduction or disease control in selected patient populations.

Moderate heterogeneity was observed (I^2^ = 50%, *p* = 0.1119), consistent with the biological and treatment diversity across studies. Importantly, the colorectal cancer study by Jonker et al. (2018) demonstrated a notable numerical benefit (HR 2.52, 95% CI: 1.44–4.41), suggesting that certain tumor types may be more responsive to reovirus-mediated oncolysis. These findings support further exploration of tumor-specific contexts and immunotherapy combinations to enhance therapeutic efficacy.

## 4. Discussion

This systematic review and meta-analysis comprehensively assessed the efficacy, safety, and biomarker-guided outcomes of oncolytic virus (OV) therapy across solid tumors. While prior studies have summarized isolated clinical effects of OV, our work uniquely integrates therapeutic outcomes with mechanistic and biomarker insights—thereby informing a precision-based approach to virotherapy.

### 4.1. Clinical Efficacy: Interpreting Patterns Beyond Statistics

Our pooled analysis confirms that OV therapies confer a modest but significant improvement in OS, PFS, and ORR. These findings reflect a tangible biological activity of OV across diverse tumor types. However, the interpretation should go beyond the statistical outputs: the most robust responses occurred in immunogenic tumors like melanoma, which already exhibit a high degree of immune infiltration and mutational burden—creating an ideal substrate for OV-mediated immunogenic killing [42,43,44]. In contrast, tumors such as pancreatic and glioblastoma, which are characterized by immune-excluded or immunosuppressive microenvironments, showed attenuated responses. This divergence suggests that OV efficacy is not solely virus-dependent but context-dependent, influenced by tumor architecture, immune accessibility, and stromal interference [84,85,86,87,88,89].

### 4.2. OV–Immune System Interplay: Explaining Therapeutic Variability

The dual mechanism of OV—direct tumor lysis and immune priming—requires a permissive host immune environment. Tumors with functional antigen presentation pathways, baseline CD8^+^ T cell infiltration, or disrupted interferon signaling are more likely to respond. The variability across trials highlights how these tumor-intrinsic features shape the downstream immune activation cascade. For example, patients with loss-of-function mutations in JAK/STAT pathways may show enhanced viral replication but insufficient immune activation unless complemented with ICIs or cytokine co-administration [90]. Therefore, outcomes observed in trials are not merely a function of viral agent selection but reflect a deeper immunological compatibility between host, tumor, and OV biology.

### 4.3. The Role of Biomarkers: From Correlation to Stratification

As oncolytic virotherapy continues to advance from experimental therapy to a more mainstream component of immuno-oncology, the identification and integration of predictive biomarkers is emerging as a cornerstone for enhancing therapeutic precision.

#### 4.3.1. Immune Biomarkers

Our analysis confirms that OV therapy reliably induces local immune activation, with multiple studies demonstrating increased CD8^+^ T-cell infiltration and elevated PD-L1 and IFN-γ expression in the tumor microenvironment [77,78,79]. These markers are well-validated, having been consistently associated with clinical responses in melanoma and other solid tumors. Peripheral shifts such as decreases in CTLA4^+^ regulatory T cells and increases in cytotoxic and memory T-cell subsets [60,81] further support systemic immune modulation, though their prognostic value requires larger prospective validation.

#### 4.3.2. Molecular/Genomic Biomarkers

Recombinant adenovirus studies [51,52,57] report upregulation of pro-apoptotic genes (p53, p21, Bax) and downregulation of angiogenic VEGF, underscoring a direct tumor-killing mechanism of OVs. While these molecular signatures reliably track virus-induced apoptosis in preclinical and early-phase trials, their translation into clinical decision-making remains speculative until correlated with patient outcomes in larger cohorts.

#### 4.3.3. Surrogate Clinical Biomarkers

Tumor burden metrics tumor size reduction and uninjected lesion responses [42,82] are robust surrogates for OV efficacy in injected and distant sites. Emerging markers such as circulating tumor cells and residual cancer burden [59,83] show promise but lack sufficient validation, as their sensitivity and specificity vary across tumor types. We have not yet identified consistent microbiome correlates of response, indicating that microbiome biomarkers are still highly exploratory.

### 4.4. OV Combinations: Clarifying Synergy and Resistance

Combination regimens involving OV and ICIs demonstrated enhanced ORRs, particularly in melanoma and KRAS-mutant NSCLC, suggesting synergistic effects [84,85]. However, not all combinations led to survival benefits—pointing toward an incomplete understanding of optimal sequencing, timing, and dose. Mechanistically, synergy depends on the OV’s ability to enhance antigen presentation and immune infiltration before the ICI takes effect. If administered too late, the immune exhaustion window may be missed [91]. Furthermore, viral neutralization by pre-existing antibodies or premature immune clearance can undermine viral propagation. Future trials must, therefore, explore immune timing kinetics, viral persistence, and co-treatment windows to maximize synergy.

### 4.5. Safety Implications: Balancing Potency and Tolerability

Across studies, OV therapy demonstrated an excellent safety profile, with most adverse events being mild flu-like symptoms or local injection-site reactions [31,90]. This favorable toxicity profile is critical for expanding OV use into frail or heavily pretreated populations. However, it also raises questions about whether current OV doses are achieving adequate intratumoral replication and immunogenicity. The balance between systemic safety and intratumoral potency is delicate—higher doses may improve efficacy but could compromise tolerability or trigger anti-viral immunity. Dose-optimization and local delivery strategies (e.g., intratumoral, intra-arterial) may offer solutions.

### 4.6. Implications for Clinical Translation and Future Directions

The findings of this meta-analysis emphasize that OV therapy holds promise as a platform for multi-modal cancer treatment, especially when integrated with biomarker-based strategies. However, its successful clinical translation hinges on several actions: designing stratified trials based on composite biomarkers (genomic, immune, microbial), employing adaptive trial frameworks that allow for cohort expansion in biomarker-enriched subsets, developing OV with payload flexibility to modulate cold tumors into immune-responsive phenotypes, expanding correlative endpoints to include serial biopsies, T cell clonality tracking, and microbiome sequencing. OV therapy is more than a viral delivery platform—it is an immunological modulator. Its full potential will only be realized when used in alignment with tumor-specific biology, immune context, and biomarker guidance. Our study provides a foundation for this transition—from empirical application toward precision virotherapy.

## 5. Conclusions

This systematic review and meta-analysis provides compelling evidence that OV therapy offers clinically meaningful benefits in the management of solid tumors, including improved ORR, modest gains in PFS, and a favorable safety profile. These outcomes support the growing role of OV as an innovative treatment modality that uniquely combines direct oncolysis with immune system activation.

Our findings underscore that the success of OV therapy is not uniform across all tumor types but is shaped by tumor immunogenicity, microenvironmental context, and the biological compatibility between the virus and host. The most notable benefits were seen in melanoma and hepatocellular carcinoma, while other malignancies such as pancreatic, colorectal, and glioblastoma exhibited more variable responses—suggesting the need for tumor-specific virotherapeutic strategies. Importantly, this study highlights the emerging role of predictive biomarkers—including tumor mutational burden, interferon signaling defects, CD8^+^ T cell infiltration, PD-L1 expression, and gut microbiota composition—in influencing response to OV therapy. Yet, current clinical trials often lack biomarker-guided patient selection, limiting therapeutic precision. Looking forward, the next frontier in OV research should prioritize biomarker-enriched trial designs, combination strategies with immune checkpoint inhibitors or targeted therapies, and the development of next-generation OV equipped with immune-stimulatory payloads. A deeper understanding of resistance mechanisms—such as antiviral immunity and immune exclusion—will also be essential for refining treatment algorithms.

In conclusion, oncolytic virotherapy represents a promising pillar in the evolving landscape of cancer immunotherapy. To fully harness its potential, future efforts must embrace personalized, mechanism-driven approaches that align viral platforms with tumor biology, immune context, and rational combination partners.

## Figures and Tables

**Table 1:** Detailed Characteristics of included Studies.

Author & Year	Tumor Type	Trial Phase/Type	Population Studied	*N* (Patients)	Intervention	Control	Primary Outcomes	Secondary Outcomes
Schenk et al., 2020 [58]	Extensive stage small cell lung cancer (ES SCLC)	Randomized, double-blind Phase II trial	ES SCLC patients who were stable/responded after platinum-based chemo	50 (26 NTX010; 24 placebo)	Single dose of NTX010 (Seneca Valley Virus)	Placebo	PFS as primary endpoint	Viral clearance, neutralizing antibody formation; OS
Heo et al., 2013 [49]	Hepatocellular carcinoma (liver cancer)	Randomized Phase 2 dose finding trial	Subjects with advanced hepatocellular carcinoma	30	Intravenous vaccinia virus JX594 (PexaVec) at low vs. high dose	Randomization between two dose levels (low dose vs. high dose)	Intrahepatic tumor responses and dose dependent effects as determined by imaging response metrics	Safety and immune stimulation endpoints (e.g., GMCSF expression)
Jonker, 2018 [53]	Metastatic colorectal cancer	Randomized Phase 2 trial	Treatment naïve patients with metastatic colorectal cancer	103 (51 per arm)	FOLFOX6/bevacizumab plus Pelareorep (oncolytic reovirus)	FOLFOX6/bevacizumab alone	Primary endpoint: PFS	Secondary endpoints: OS, ORR, quality of life
Kaufman hl et al., 2016 [69]	Advanced melanoma (stage IIIC/IV)	Multi-institutional Phase 2 single arm trial	Patients with stage IIIC or IV melanoma (treated by TVEC)	50	Intra-lesional TVEC	None (all patients received TVEC; analysis compared injected vs. non injected lesions)	Lesion response rates in both directly injected and uninjected lesions; assessment of systemic effect via lesion shrinkage	Time to response (median response time per lesion type)
Malvehy et al., 2021 [70]	Melanoma (stage IIIB–IVM1c)	Phase 2 multicenter, open label study	Patients with unresectable melanoma (stage IIIB–IVM1c)	112 enrolled (111 treated; subset available for biomarker analysis)	Intralesional TVEC	None (single arm study)	Objective response rates (e.g., overall response: 28% and complete response: 14% overall; 32%/18% in stage IIIB–IVM1a subgroup) and evaluation of immune cell density changes in noninjected lesions	Exploratory analyses of changes in specific immune-cell subsets in noninjected lesions
Moehler et al., 2019 [50]	Advanced HCC	Phase IIb, randomized, multicenter trial (TRAVERSE)	Patients with advanced HCC following sorafenib failure	Not specified	Vaccinia based oncolytic immunotherapy Pexastimogene Devacirepvec (PexaVec)	Control arm (standard care/control treatment)	Efficacy endpoints (e.g., OS, ORR, disease control) and safety	Secondary outcomes: PFS, safety, and immune endpoints
Noonan et al., 2016 [60]	metastatic pancreatic adenocarcinoma	Randomized Phase 2 trial	Treatment naïve patients with metastatic pancreatic adenocarcinoma	73 evaluable (36 in Arm A, 37 in Arm B)	Combination of carboplatin/paclitaxel with pelareorep (oncolytic reovirus) (Arm A)	Carboplatin/paclitaxel alone (Arm B)	Primary endpoint: PFS	Secondary endpoints: OS, ORR, and assessment of immunologic biomarkers (cytokine levels, immune cell subsets)
Andtbacka rhi et al. (2016) [42]	Unresectable stage IIIB–IV melanoma	Phase III (OPTiM trial; post hoc lesion-level analysis)	Patients with unresectable melanoma treated with T-VEC	(*N* = 295 in T-VEC arm)	Intralesional TVEC	Subcutaneous GMCSF (in OPTiM)	Durable response rate; lesion level reduction of ≥50% in size	Analysis of patterns (e.g., appearance of new lesions or ≥25% increase before response [PPR]); correlation with OS
Bernstein et al. (2018) [59]	Metastatic breast cancer	Randomized Phase II	Women with previously treated metastatic breast cancer	*N* = 74	Weekly paclitaxel plus pelareorep (oncolytic reovirus)	Weekly paclitaxel alone	Primary endpoint: PFS	Secondary endpoints: OS, ORR, CTC counts, safety
Bradbury et al. (2018) [54]	Advanced/metastatic non-small cell lung cancer (NSCLC)	Randomized Phase II	Patients with advanced/metastatic NSCLC after first-line chemotherapy	*N* = 166	Second line chemotherapy (pemetrexed [for nonsquamous] or docetaxel [for squamous]) plus pelareorep	Chemotherapy alone	Primary endpoint: PFS	Secondary endpoints: OS, ORR, exploratory translational analyses
Chesney et al. (2018) [45]	Advanced, unresectable melanoma	Randomized Phase II (open-label)	Patients with advanced melanoma	(*N* = 198; 98 in combination arm, 100 in ipilimumab arm)	Intralesional TVEC in combination with ipilimumab	Ipilimumab alone	Primary endpoint: ORR by immune related response criteria	Secondary endpoints: Lesion responses (including responses in uninjected lesions) and safety
Chesney ja et al. (2023) [41]	Advanced unresectable melanoma (stage IIIB–IVM1c)	Phase III, randomized, double-blind, placebo-controlled	Patients with unresectable melanoma	(PD-1 naïve; total *N* = 692, with 346 per arm)	Intralesional TVEC plus pembrolizumab	Placebo plus pembrolizumab	Dual primary endpoints: PFS (per mRECIST by blinded independent review) and OS	Secondary endpoints: ORR, CRR, durable response rate, safety
Cohn et al. (2017) [61]	Recurrent or persistent epithelial ovarian, tubal, or peritoneal carcinoma	Randomized Phase IIB	Women with recurrent or persistent ovarian, tubal, or peritoneal cancer	(*N* = 108 evaluable)	Weekly paclitaxel plus oncolytic reovirus (Reolysin)	Weekly paclitaxel alone	Primary endpoint: PFS	Secondary endpoints: ORR, toxicity, and correlative biomarker analyses
Freytag et al. (2014) [63]	Intermediate risk prostate cancer	Prospective Randomized Phase II	Men with intermediate-risk prostate cancer	*N* = 44; 21 in experimental arm, 23 in control	Intensity modulated radiation therapy (IMRT) combined with oncolytic adenovirus-mediated cytotoxic gene therapy (OAMCGT)	IMRT only	Primary endpoint: Acute toxicity (<90 days) and 2-year prostate biopsy positivity	Secondary endpoints: Quality of life (QOL), freedom from biochemical/clinical failure, freedom from metastases, survival
Galanis et al. (2012) [71]	Metastatic melanoma	Phase II, single-arm	Patients with metastatic melanoma	21	Intravenous administration of Reolysin^®^ (3 × 10^10^ TCID50 on days 1–5 every 28 days)	None	Primary endpoint: Antitumor effect (clinical benefit rate) and toxicity profile	Secondary endpoints: Median time to progression (45 days) and median OS (165 days)
Lindsey et al., 2006 [72]	Metastatic melanoma	Randomized Phase II (three-arm design plus a sequential cohort trial)		64	Recombinant poxvirus vaccines (vaccinia and fowlpox vectors) encoding full-length tyrosinase; administered either alone or with concurrent (low or high dose) IL2, or sequential high dose IL2 in the second trial	Vaccine-alone arm served as comparator within the trial	Objective clinical response (tumor shrinkage) and immunologic response (measured in vitro)	Assessment of lesional regression (mixed and minor responses)
Voit et al., 2003 [73]	Resectable Stage III melanoma (adjuvant setting)	Phase II trial (adjuvant immunotherapy)	Patients with melanoma	20 patients	Intradermal injection of NDV-modified autologous melanoma cell lysate combined with interleukin2	Matched-pair control (patients managed with surgery alone)	Induction of DTH response and reduction in tumor recurrence rate	Safety/tolerability and immunologic profile
Habib et al., 2002 [48]	Hepatocellular carcinoma (HCC)	Open-label, randomized Phase I/II trial	Patients with HCC	10 patients	E1Bdeleted adenovirus (dl1520) gene therapy for HCC	Percutaneous ethanol injection (PEI)	Tumor response (partial response vs. progression) and safety	Evaluation of toxicity and complications
Wallack et al., 1995 [46]	Stage II melanoma (surgical adjuvant setting)	Phase III randomized, double-blind, multi-institutional trial	Patients with melanoma	250 patients (104 VMO; 113 placebo)	Vaccinia melanoma oncolysate (VMO) vaccine administered post-surgery	Placebo vaccinia virus (identical virus given as control)	Increase in DFI and OS in the adjuvant setting	Immunologic evaluation (DTH skin test) and safety/tolerability
Wallack et al., 1997 [47]	Stage II melanoma (surgical adjuvant setting)	Phase III randomized multi-institutional trial—Second interim analysis	Patients with melanoma	Subset details from same trial as [46]	Vaccinia melanoma oncolysate (VMO) vaccine	Placebo vaccinia virus	Primary endpoints: OS and DFS	Retrospective subset analyses to identify groups with survival benefit
Schlag et al., 1992 [39]	Colorectal cancer with liver metastases	Phase II trial (adjuvant active specific immunotherapy)	Patients with Colorectal cancer	23 patients	Autologous, irradiated tumor cell vaccine incubated with 32 HU Newcastle disease virus (NDV); administered intradermally	Matched-pair control (surgery only)	Primary endpoints: DTH response enhancement and reduction in tumor recurrence rate	Safety and immunologic tolerance; comparison of DTH to standard antigens
Chesney et al., 2023 [40]	Advanced unresectable melanoma	Multicenter, randomized, open-label Phase II trial	Patients with melanoma	198 patients (98 in T-VEC plus ipilimumab arm; 100 in ipilimumab alone arm)	Intralesional TVEC combined with intravenous ipilimumab (3 mg/kg, up to 4 doses)	Ipilimumab alone	Primary endpoint: Investigator-assessed ORR per immune-related criteria	Secondary endpoints: DRR, duration of response (DOR), median PFS, OS, and safety
Villalona-calero et al., 2016 [55]	Metastatic/recurrent NSCLC with KRAS-activated tumors	Phase II trial	Patients with metastatic/recurrent NSCLC harboring Ras pathway activation	37	Paclitaxel + carboplatin + Reolysin^®^ (IV)	No formal control (single-arm study)	Primary: Tumor response (RECIST) and tolerability	Secondary: OS exploratory biomarker analyses
Lu et al., 2004 [74]	Advanced refractory malignant tumors (various solid tumors)				Intratumoral H101 (recombinant adenovirus) + standard chemotherapy	Internal control: Non injected lesions in the same patients	Primary: Objective response in injected lesions	Secondary: Comparison of non-injected lesion responses, safety
Khuri et al., 2000 [66]	Recurrent squamous cell carcinoma of the head and neck	Phase II controlled trial	Patients with recurrent head and neck SCC	37 enrolled; 30 evaluable for response	Intratumoral ONYX-015 + IV cisplatin and 5fluorouracil	Intrapatient control: Non injected tumor masses (treated with chemotherapy alone)	Primary: Tumor responses (injected lesions)	Secondary: Duration of response, safety/tolerability, demonstration of viral replication
Ma et al., 2017 [56]	Recurrent nasopharyngeal carcinoma (NPC)	Randomized Phase II trial	Patients with recurrent NPC; 162 total (54 per treatment arm across 3 arms)	162 (54 in each of 3 groups)	Recombinant human adenovirus p53 (rAdp53) + chemoradiotherapy (CRT)	Two control groups: CRT alone and rAdp53 alone	Primary: Objective response rate (CR + PR) by RECIST	Secondary: Changes in serum tumor markers, toxicity, PFS, and 3-year survival rate
Harrington et al., 2016 [75]	Unresectable stage IIIB–IVM1a melanoma (OPTiM subgroup)	Subgroup analysis from a Phase III trial	Patients with stage IIIB–IVM1a melanoma; 249 patients in subgroup	249 (from OPTiM subgroup)	Intralesional TVEC (administered per protocol)	Subcutaneous recombinant GMCSF	Primary: DRR and ORR in stage IIIB–IVM1a melanoma	Secondary: CR rate, duration of response, and safety
Kelly et al., 2020 [64]	Locally advanced or metastatic sarcoma	Phase 2, single-institution open-label trial	Patients with advanced sarcoma refractory to standard systemic therapy	20	Intratumoral TVEC (4 mL; first dose at 10^6^ PFU/mL then 10^8^ PFU/mL) + intravenous pembrolizumab (200 mg flat dose every 3 weeks)	None (single arm)	Primary: ORR at 24 weeks by RECIST	Secondary: Best ORR by immune-related RECIST, PFS, OS, safety
Eigl et al., 2018 [62]	Metastatic castration-resistant prostate cancer (mCRPC)	Randomized Phase II trial	Men with mCRPC	85	Docetaxel (75 mg/m^2^ IV on day 1 every 21-day cycle) + pelareorep (Reolysin^®^)	Docetaxel (75 mg/m^2^ IV on day 1 every 21-day cycle) + prednisone 5 mg twice daily	Primary: 12-week lack of disease progression (LPD rate)	Secondary: OS
Cerullo et al. (2011) [76]	Advanced solid tumors (refractory)	Phase I/II (non-randomized)	Patients with Advanced solid tumors (refractory)	42 vs. 8	Oncolytic adenovirus + low-dose cyclophosphamide	Oncolytic adenovirus alone	Safety, Treg reduction	PFS, OS, disease control rate
Yang et al. (2010) [52]	Hepatocellular carcinoma (HCC)	Phase II (RCT)	Patients with HCC	20 vs. 20	rAd-p53 + fractionated stereotactic RT (fSRT)	fSRT alone	Tumor response, survival rates	Toxicity
Tian et al. (2009) [51]	Unresectable HCC	Pilot Phase II (RCT)	Patients with HCC	23 vs. 23	rAd-p53 + 5-FU after TACE	TACE alone	Efficacy (TTP, OS)	Toxicity, biomarker expression
Pan et al. (2009) [57]	Nasopharyngeal carcinoma (NPC)	Phase II (RCT)	Patients with NPC	42 vs. 40	rAd-p53 + RT	RT alone	Loco regional control, survival	Toxicity
Dong et al. (2008) [68]	Malignant pleural/peritoneal effusion (various cancers)		Patients different cancers	27 vs. 21	rAd-p53 + cisplatin	Cisplatin alone	Effusion control, Karnofsky score improvement	Toxicity
Xing Su et al. (2016) [67]	Locally Advanced Cervical Cancer	Randomized Controlled Phase 2	Patients with Locally Advanced Cervical Cancer	69 (PRT) vs. 35 (RT)	rAd-p53 + Radiotherapy	Radiotherapy alone	Safety 5-year OS and PFS	Loco regional recurrence rate Distant metastasis rate
Manfred Westphal et al. (2013) [65]	Glioblastoma Multiforme	Phase 3 (ASPECT Trial)	Patients with Glioblastoma Multiforme	124 (sitimagene) vs. 126 (standard)	Sitimagene ceradenovec + ganciclovir	Standard care (surgery + radiotherapy)	Time to death or re-intervention (composite endpoint)	OS Safety

**Table 2:** Biomarker Changes During OV Therapy (Monotherapy and Combination).

Biomarker Category	Specific Biomarker	Change During Treatment	Reported In
Immune Cell Infiltration	CD8^+^ T cells	↑	Ribas et al. (2017), Shoushtari et al. (2023), Schwarze et al. (2022) [77,78,79]
	PD-L1 expression	↑	Ribas et al. (2017), Schwarze et al. (2022) [77,78]
	IFN-γ gene expression	↑	Shoushtari et al. (2023), Schwarze et al. (2022) [78,79]
Viral Biomarkers	Viral replication	↑	Schenk et al. (2020), Garcia et al. (2018) [58,80]
	Neutralizing antibodies	↑	Schenk et al. (2020), Garcia et al. (2018) [58,80]
Cytokines & Peripheral Markers	CTLA4^+^ Tregs	↓	Noonan et al. (2016), Mahalingam et al. (2020) [60,81]
	Cytotoxic T cells (e.g., CD8^+^)	↑	Noonan et al. (2016), Mahalingam et al. (2020) [60,81]
	Memory T-cell subsets	↑	Mahalingam et al. (2020) [81]
Genetic/Molecular Signatures	p53 mRNA, p21, Bax (pro-apoptotic)	↑	Yang et al. (2010), Tian et al. (2009), Pan et al. (2009) [51,52,57]
	VEGF (angiogenesis marker)	↓	Yang et al. (2010), Pan et al. (2009) [52,57]
Tumor Burden Surrogates	Tumor size/volume	↓	Andtbacka et al. (2016), Stahlie et al. (2021) [42,82]
	Lesion-level response (uninjected tumor)	↑	Andtbacka et al. (2016), Stahlie et al. (2021) [42,82]
Surrogate Clinical Biomarkers	Circulating Tumor Cells (CTCs)	↓	Bernstein et al. (2018), Soliman et al. (2020) [59,83]
	Residual Cancer Burden (RCB)	↓	Bernstein et al. (2018), Soliman et al. (2020) [59,83]

Arrows indicate direction of change during treatment: ↑ = increase during treatment, ↓ = decrease during treatment.

## Data Availability

The original contributions presented in this study are included in this article/Supplementary Materials. Further inquiries can be directed to the corresponding author.

## Supplementary Materials

The following supporting information can be downloaded at: https://www.mdpi.com/article/10.3390/vaccines13101070/s1. Supplementary Figure S1: Study Selection as per PRISMA 2020 flow diagram [92], Supplementary S1: Search Strategy.
