# Supplementary material for: Efficacy, Safety and Predictive Biomarkers of Oncolytic Virus Therapy in Solid Tumors: A Systematic Review and Meta-Analysis
Source: Vaccines (Basel). 2025 Oct 20;13(10):1070. doi: 10.3390/vaccines13101070 (PMC12567567; doi:10.3390/vaccines13101070)
Supplement: Supplementary file 1 [file vaccines-13-01070-s001.zip › vaccines-3714800-supplementary.pdf]

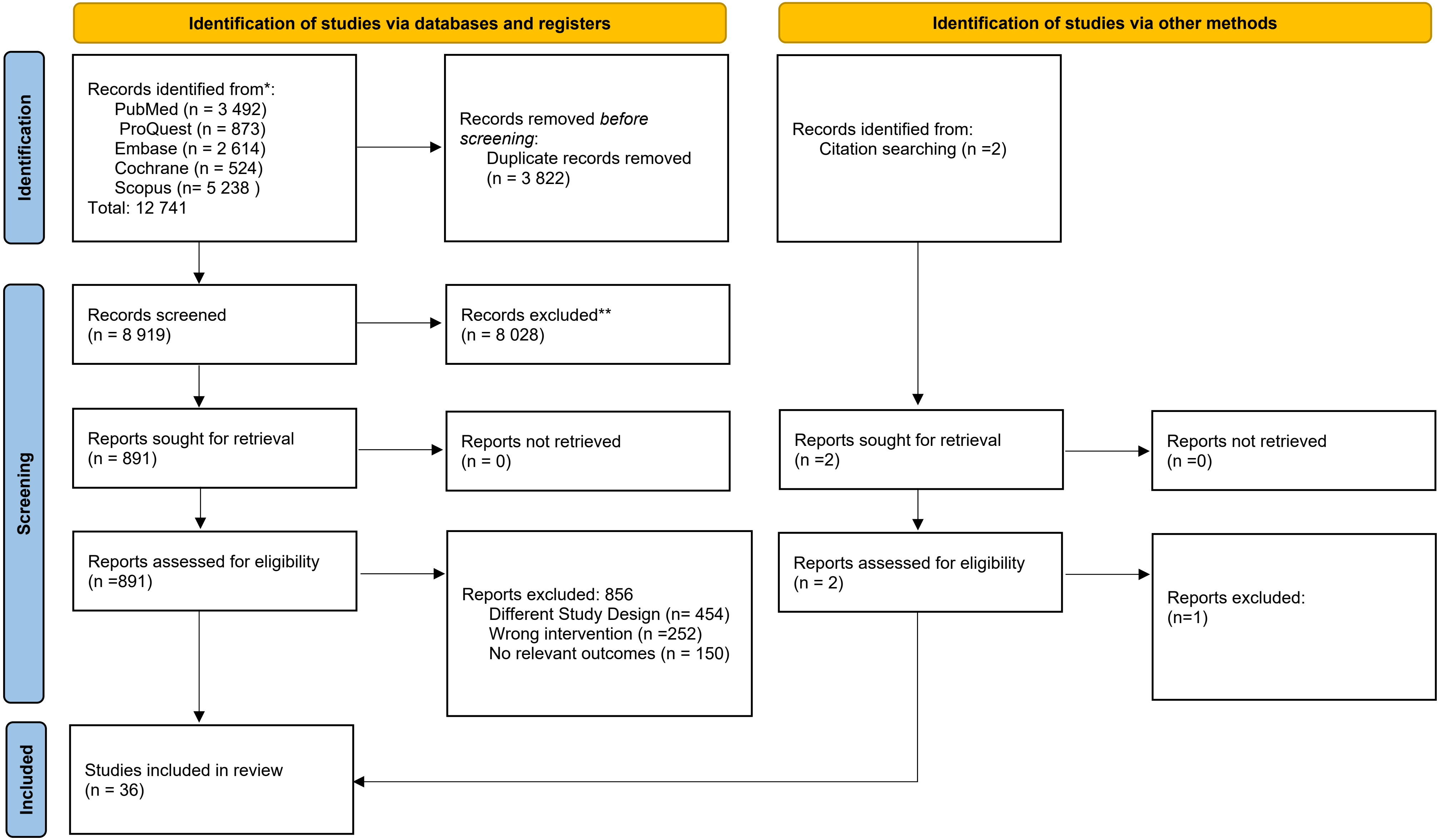

Supplementary Figure: 1 Study Selection as per PRISMA 2020

*Consider, if feasible to do so, reporting the number of records identified from each database or register searched (rather than the total number across all databases/registers).
**If automation tools were used, indicate how many records were excluded by a human and how many were excluded by automation tools.

From: Page MJ, McKenzie JE, Bossuyt PM, Boutron I, Hoffmann TC, Mulrow CD, et al. The PRISMA 2020 statement: an updated guideline for reporting systematic reviews. BMJ 2021;372:n71. doi: 10.1136/bmj.n71. For more information, visit: http://www.prisma-statement.org/

Search Strategy

A1. PubMed (MEDLINE via PubMed)

Limits: Humans; English; 2000–2025

- 1. "Oncolytic Viruses"[Mesh] OR oncolytic virus*[tiab] OR talimogene laherparepvec[tiab] OR T-VEC[tiab] OR reovirus[tiab] OR adenovirus[tiab] OR vaccinia[tiab] OR "measles virus"[tiab]

2. "Neoplasms"[Mesh] OR cancer*[tiab] OR tumor*[tiab] OR tumour*[tiab] OR melanoma[tiab] OR lymphoma[tiab] OR leukemia[tiab]

3. "Biological Markers"[Mesh] OR biomarker*[tiab] OR "tumor mutational burden"[tiab] OR TMB[tiab] OR PD-L1[tiab] OR microbiome[tiab]

4. clinical trial[pt] OR randomized controlled trial[pt] OR random*[tiab] OR cohort[tiab] OR observational[tiab] OR trial[tiab]

- 5. Combine: #1 AND #2 AND (#3 OR #4)

A2. Embase

Limits: Humans; English; 2000–2025

- 1. 'oncolytic virus'/exp OR oncolytic virus*:ti,ab OR talimogene laherparepvec:ti,ab OR T-VEC:ti,ab OR reovirus:ti,ab OR adenovirus:ti,ab OR vaccinia:ti,ab OR measles virus:ti,ab

2. 'neoplasm'/exp OR neoplasm*:ti,ab OR tumor*:ti,ab OR tumour*:ti,ab OR cancer*:ti,ab OR melanoma:ti,ab OR lymphoma:ti,ab OR leukemia:ti,ab

3. 'biomarker'/exp OR biomarker*:ti,ab OR 'tumor mutational burden':ti,ab OR TMB:ti,ab OR PD-L1:ti,ab OR microbiome:ti,ab

4. random*:ti,ab OR 'clinical trial'/de OR cohort:ti,ab OR observational:ti,ab OR trial:ti,ab

- 5. Combine: 1 AND 2 AND (3 OR 4)

A3. Cochrane CENTRAL

Limits: English; 2000–2025

- 1. MeSH descriptor: [Oncolytic Viruses] explode all trees

2. oncolytic virus* OR talimogene laherparepvec OR T-VEC OR reovirus OR adenovirus OR vaccinia OR measles virus

3. MeSH descriptor: [Neoplasms] explode all trees OR neoplasm* OR tumor* OR tumour* OR cancer* OR melanoma OR lymphoma OR leukemia

4. MeSH descriptor: [Biological Markers] explode all trees OR biomarker* OR tumor mutational burden OR TMB OR PD-L1 OR microbiome

- 5. Combine: (#1 OR #2) AND (#3 OR #4)

A4. Scopus
Limits: English; 2000–2025

- TITLE-ABS-KEY("oncolytic virus" OR "T-VEC" OR talimogene laherparepvec OR reovirus OR adenovirus OR vaccinia OR "measles virus")
- AND TITLE-ABS-KEY(cancer OR tumor OR tumour OR neoplasm OR melanoma OR lymphoma OR leukemia)
- AND TITLE-ABS-KEY(biomarker OR "tumor mutational burden" OR TMB OR PD-L1 OR microbiome OR random* OR trial OR cohort OR observational)
